# Supplementary material for: Microbial communities associated with the black morel Morchella sextelata cultivated in greenhouses
Source: PeerJ. 2019 Sep 26;7:e7744. doi: 10.7717/peerj.7744 (PMC6766373; doi:10.7717/peerj.7744)
Supplement: Supplemental Information 1 [file peerj-07-7744-s001.pdf]

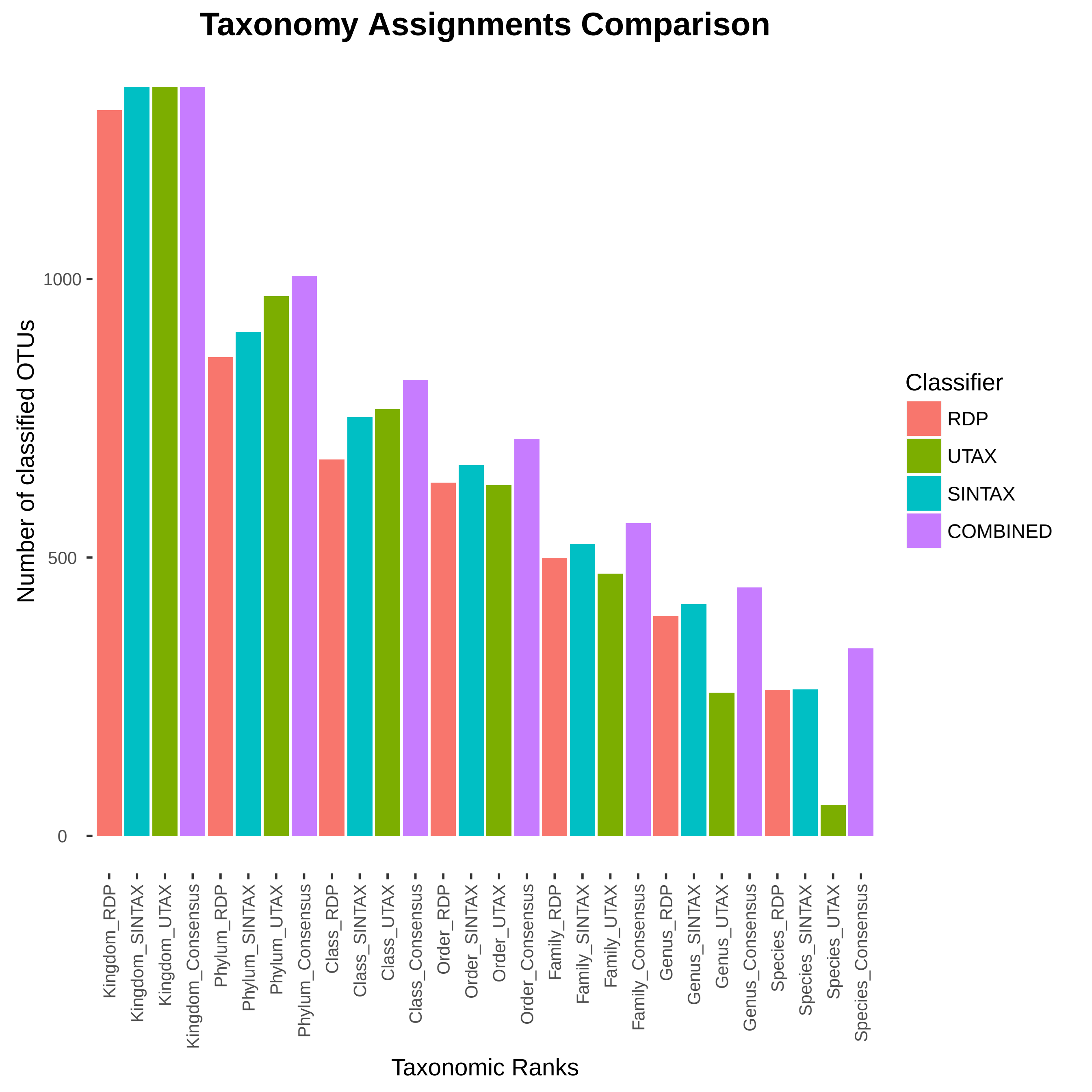
Taxonomy Assignments Comparison
Number of classified OTUs
1000
500
0
Kingdom_RDP
Kingdom_SINTAX
Kingdom_UTAX
Kingdom_Consensus
Phylum_RDP
Phylum_SINTAX
Phylum_UTAX
Phylum_Consensus
Class_RDP
Class_SINTAX
Class_UTAX
Class_Consensus
Order_RDP
Order_SINTAX
Order_UTAX
Order_Consensus
Family_RDP
Family_SINTAX
Family_UTAX
Family_Consensus
Genus_RDP
Genus_SINTAX
Genus_UTAX
Genus_Consensus
Species_RDP
Species_SINTAX
Species_UTAX
Species_Consensus
Taxonomic Ranks
Classifier
RDP
UTAX
SINTAX
COMBINED
