# Supplementary figures and images for: Microbial communities associated with the black morel Morchella sextelata cultivated in greenhouses
Source: PeerJ. 2019 Sep 26;7:e7744. doi: 10.7717/peerj.7744 (PMC6766373; doi:10.7717/peerj.7744)

# Prokaryotes

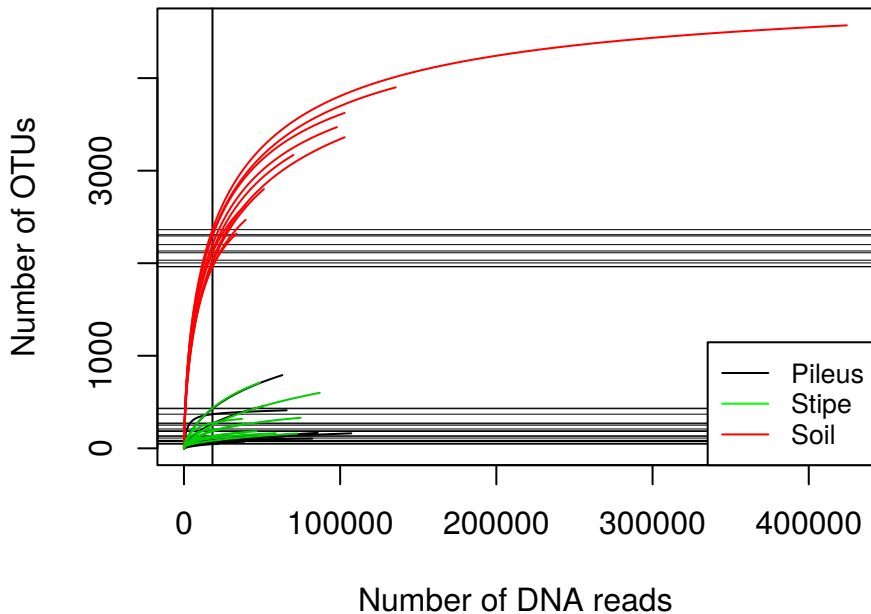

Supplement: Supplemental Information 2 [file peerj-07-7744-s002.pdf]

# Fungi

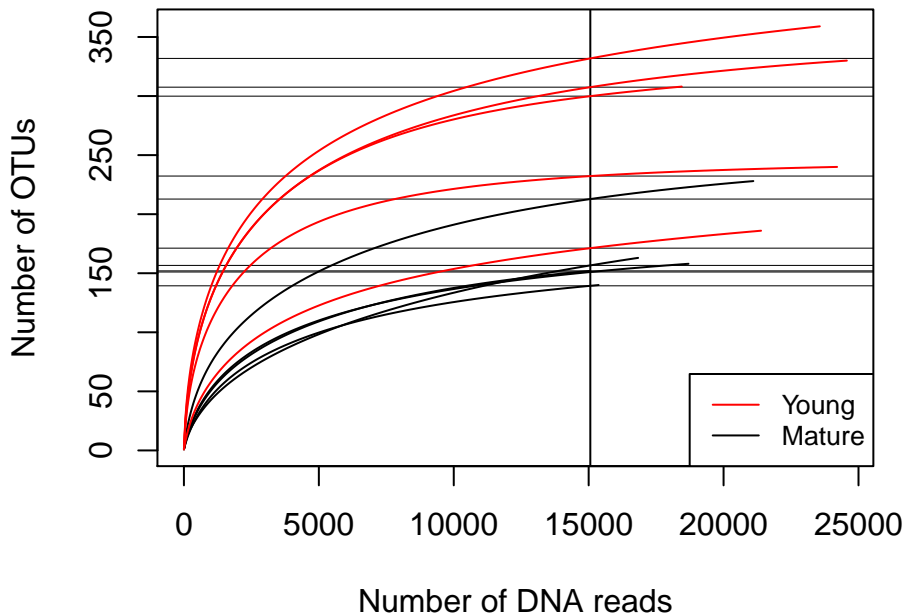

Supplement: Supplemental Information 3 [file peerj-07-7744-s003.pdf]

## (A) Prokaryotes

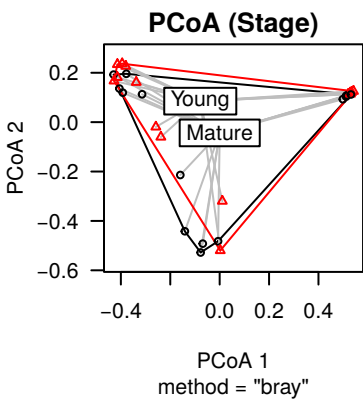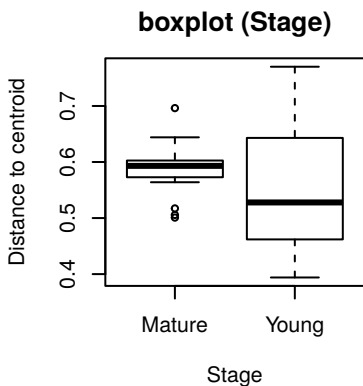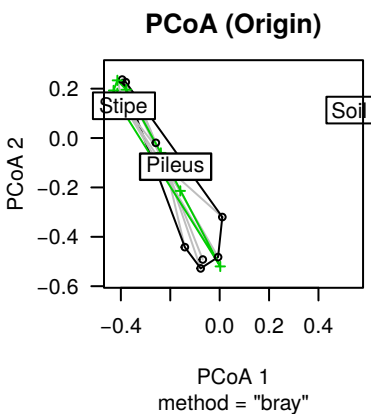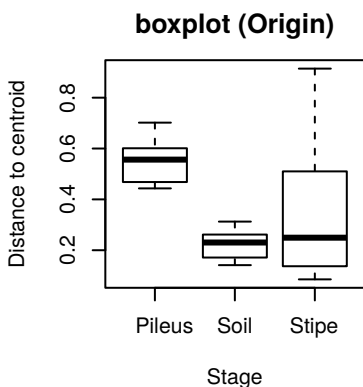

## (B) Fungi

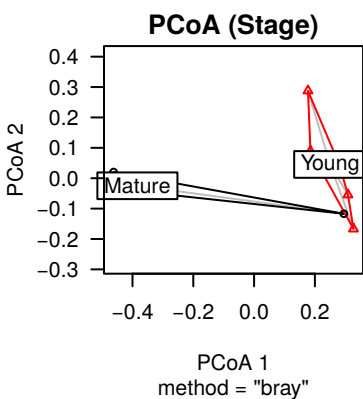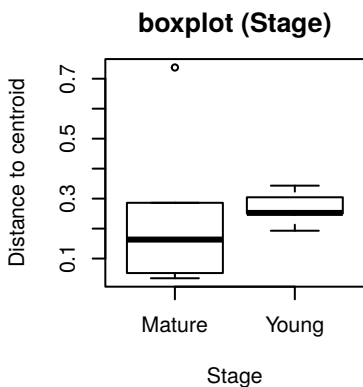

Supplement: Supplemental Information 4 — From left to right: principal coordinate analysis (PCoA) ordinations according “Origin” and “Stage” (only for prokaryotes) of Bray Curtis dissimilarities and boxplot showing distribution of distances form group centroids. [file peerj-07-7744-s004.pdf]
