# Supplementary material for: Microbial communities associated with the black morel Morchella sextelata cultivated in greenhouses
Source: PeerJ. 2019 Sep 26;7:e7744. doi: 10.7717/peerj.7744 (PMC6766373; doi:10.7717/peerj.7744)
Supplement: Supplemental Information 5 [file peerj-07-7744-s005.docx]

|  |
| --- |
| Multilevel pattern analysis |
| --------------------------- |
|  |
| Association function: IndVal.g |
| Significance level (alpha): 0.05 |
|  |
| Total number of species: 5169 |
| Selected number of species: 3768 |
| Number of species associated to 1 group: 3743 |
| Number of species associated to 2 groups: 25 |
|  |
| List of species associated to each combination: |
|  |
| Group Pileus #sps. 2 |
| stat p.value |
| OTU_6929 0.767 0.0308 * |
| OTU_1794 0.764 0.0207 * |
|  |
| Group Soil #sps. 3739 |
| stat p.value |
| OTU_869 1.000 0.000253 *** |
| OTU_94 1.000 0.000253 *** |
| OTU_785 1.000 0.000253 *** |
| OTU_1229 1.000 0.000253 *** |
| OTU_204 1.000 0.000253 *** |
| OTU_10659 1.000 0.000253 *** |
| OTU_818 1.000 0.000253 *** |
| OTU_2777 1.000 0.000253 *** |
| OTU_285 1.000 0.000253 *** |
| OTU_2790 1.000 0.000253 *** |
| OTU_870 1.000 0.000253 *** |
| OTU_3177 1.000 0.000253 *** |
| OTU_602 1.000 0.000253 *** |
| OTU_8712 1.000 0.000253 *** |
| OTU_3165 1.000 0.000253 *** |
| OTU_3098 1.000 0.000253 *** |
| OTU_383 1.000 0.000253 *** |
| OTU_498 1.000 0.000253 *** |
| OTU_11226 1.000 0.000253 *** |
| OTU_982 1.000 0.000253 *** |
| OTU_90 1.000 0.000253 *** |
| OTU_2749 1.000 0.000253 *** |
| OTU_4991 1.000 0.000253 *** |
| OTU_12181 1.000 0.000253 *** |
| OTU_610 1.000 0.000253 *** |
| OTU_284 1.000 0.000253 *** |
| OTU_2186 1.000 0.000253 *** |
| OTU_1958 1.000 0.000253 *** |
| OTU_462 1.000 0.000253 *** |
| OTU_725 1.000 0.000253 *** |
| OTU_380 1.000 0.000253 *** |
| OTU_325 1.000 0.000253 *** |
| OTU_3095 1.000 0.000253 *** |
| OTU_319 1.000 0.000253 *** |
| OTU_1703 1.000 0.000253 *** |
| OTU_674 1.000 0.000253 *** |
| OTU_2112 1.000 0.000253 *** |
| OTU_2430 1.000 0.000253 *** |
| OTU_7130 1.000 0.000253 *** |
| OTU_6552 1.000 0.000253 *** |
| OTU_938 1.000 0.000253 *** |
| OTU_396 1.000 0.000253 *** |
| OTU_300 1.000 0.000253 *** |
| OTU_477 1.000 0.000253 *** |
| OTU_4313 1.000 0.000253 *** |
| OTU_925 1.000 0.000253 *** |
| OTU_2783 1.000 0.000253 *** |
| OTU_995 1.000 0.000253 *** |
| OTU_4468 1.000 0.000253 *** |
| OTU_237 1.000 0.000253 *** |
| OTU_392 1.000 0.000253 *** |
| OTU_515 1.000 0.000253 *** |
| OTU_2631 1.000 0.000253 *** |
| OTU_1175 1.000 0.000253 *** |
| OTU_1150 1.000 0.000253 *** |
| OTU_1180 1.000 0.000253 *** |
| OTU_968 1.000 0.000253 *** |
| OTU_1118 1.000 0.000253 *** |
| OTU_520 1.000 0.000253 *** |
| OTU_423 1.000 0.000253 *** |
| OTU_535 1.000 0.000253 *** |
| OTU_506 1.000 0.000253 *** |
| OTU_3608 1.000 0.000253 *** |
| OTU_12218 1.000 0.000253 *** |
| OTU_1481 1.000 0.000253 *** |
| OTU_762 1.000 0.000253 *** |
| OTU_2623 1.000 0.000253 *** |
| OTU_2356 1.000 0.000253 *** |
| OTU_253 1.000 0.000253 *** |
| OTU_1291 1.000 0.000253 *** |
| OTU_836 1.000 0.000253 *** |
| OTU_666 1.000 0.000253 *** |
| OTU_433 1.000 0.000253 *** |
| OTU_4791 1.000 0.000253 *** |
| OTU_3149 1.000 0.000253 *** |
| OTU_2218 1.000 0.000253 *** |
| OTU_9669 1.000 0.000253 *** |
| OTU_451 1.000 0.000253 *** |
| OTU_1319 1.000 0.000253 *** |
| OTU_2174 1.000 0.000253 *** |
| OTU_763 1.000 0.000253 *** |
| OTU_2850 1.000 0.000253 *** |
| OTU_3352 1.000 0.000253 *** |
| OTU_2221 1.000 0.000253 *** |
| OTU_1664 1.000 0.000253 *** |
| OTU_8368 1.000 0.000253 *** |
| OTU_1469 1.000 0.000253 *** |
| OTU_2129 1.000 0.000253 *** |
| OTU_9292 1.000 0.000253 *** |
| OTU_2052 1.000 0.000253 *** |
| OTU_2366 1.000 0.000253 *** |
| OTU_1782 1.000 0.000253 *** |
| OTU_1159 1.000 0.000253 *** |
| OTU_1519 1.000 0.000253 *** |
| OTU_1139 1.000 0.000253 *** |
| OTU_1421 1.000 0.000253 *** |
| OTU_4593 1.000 0.000253 *** |
| OTU_1059 1.000 0.000253 *** |
| OTU_1612 1.000 0.000253 *** |
| OTU_1233 1.000 0.000253 *** |
| OTU_3355 1.000 0.000253 *** |
| OTU_920 1.000 0.000253 *** |
| OTU_634 1.000 0.000253 *** |
| OTU_672 1.000 0.000253 *** |
| OTU_7640 1.000 0.000253 *** |
| OTU_1665 1.000 0.000253 *** |
| OTU_4830 1.000 0.000253 *** |
| OTU_3749 1.000 0.000253 *** |
| OTU_1326 1.000 0.000253 *** |
| OTU_772 1.000 0.000253 *** |
| OTU_10373 1.000 0.000253 *** |
| OTU_1038 1.000 0.000253 *** |
| OTU_264 1.000 0.000253 *** |
| OTU_534 1.000 0.000253 *** |
| OTU_3727 1.000 0.000253 *** |
| OTU_4898 1.000 0.000253 *** |
| OTU_2745 1.000 0.000253 *** |
| OTU_1281 1.000 0.000253 *** |
| OTU_1328 1.000 0.000253 *** |
| OTU_809 1.000 0.000253 *** |
| OTU_750 1.000 0.000253 *** |
| OTU_1016 1.000 0.000253 *** |
| OTU_3121 1.000 0.000253 *** |
| OTU_2906 1.000 0.000253 *** |
| OTU_355 1.000 0.000253 *** |
| OTU_3524 1.000 0.000253 *** |
| OTU_2271 1.000 0.000253 *** |
| OTU_707 1.000 0.000253 *** |
| OTU_840 1.000 0.000253 *** |
| OTU_2019 1.000 0.000253 *** |
| OTU_1321 1.000 0.000253 *** |
| OTU_7859 1.000 0.000253 *** |
| OTU_8601 1.000 0.000253 *** |
| OTU_531 1.000 0.000253 *** |
| OTU_1554 1.000 0.000253 *** |
| OTU_9457 1.000 0.000253 *** |
| OTU_7415 1.000 0.000253 *** |
| OTU_4566 1.000 0.000253 *** |
| OTU_916 1.000 0.000253 *** |
| OTU_11475 1.000 0.000253 *** |
| OTU_2535 1.000 0.000253 *** |
| OTU_408 1.000 0.000253 *** |
| OTU_4268 1.000 0.000253 *** |
| OTU_1455 1.000 0.000253 *** |
| OTU_867 1.000 0.000253 *** |
| OTU_2180 1.000 0.000253 *** |
| OTU_759 1.000 0.000253 *** |
| OTU_2983 1.000 0.000253 *** |
| OTU_9196 1.000 0.000253 *** |
| OTU_9400 1.000 0.000253 *** |
| OTU_2256 1.000 0.000253 *** |
| OTU_2929 1.000 0.000253 *** |
| OTU_618 1.000 0.000253 *** |
| OTU_651 1.000 0.000253 *** |
| OTU_1970 1.000 0.000253 *** |
| OTU_1231 1.000 0.000253 *** |
| OTU_1052 1.000 0.000253 *** |
| OTU_4002 1.000 0.000253 *** |
| OTU_779 1.000 0.000253 *** |
| OTU_8120 1.000 0.000253 *** |
| OTU_2879 1.000 0.000253 *** |
| OTU_5578 1.000 0.000253 *** |
| OTU_1301 1.000 0.000253 *** |
| OTU_2438 1.000 0.000253 *** |
| OTU_326 1.000 0.000253 *** |
| OTU_880 1.000 0.000253 *** |
| OTU_2069 1.000 0.000253 *** |
| OTU_1185 1.000 0.000253 *** |
| OTU_1674 1.000 0.000253 *** |
| OTU_1747 1.000 0.000253 *** |
| OTU_1903 1.000 0.000253 *** |
| OTU_926 1.000 0.000253 *** |
| OTU_2512 1.000 0.000253 *** |
| OTU_2133 1.000 0.000253 *** |
| OTU_3776 1.000 0.000253 *** |
| OTU_3771 1.000 0.000253 *** |
| OTU_2499 1.000 0.000253 *** |
| OTU_3054 1.000 0.000253 *** |
| OTU_2254 1.000 0.000253 *** |
| OTU_1838 1.000 0.000253 *** |
| OTU_2336 1.000 0.000253 *** |
| OTU_2014 1.000 0.000253 *** |
| OTU_5850 1.000 0.000253 *** |
| OTU_1262 1.000 0.000253 *** |
| OTU_1704 1.000 0.000253 *** |
| OTU_1807 1.000 0.000253 *** |
| OTU_1666 1.000 0.000253 *** |
| OTU_2113 1.000 0.000253 *** |
| OTU_2923 1.000 0.000253 *** |
| OTU_556 1.000 0.000253 *** |
| OTU_1956 1.000 0.000253 *** |
| OTU_11282 1.000 0.000253 *** |
| OTU_6659 1.000 0.000253 *** |
| OTU_519 1.000 0.000253 *** |
| OTU_9186 1.000 0.000253 *** |
| OTU_3218 1.000 0.000253 *** |
| OTU_1649 1.000 0.000253 *** |
| OTU_7884 1.000 0.000253 *** |
| OTU_2608 1.000 0.000253 *** |
| OTU_2241 1.000 0.000253 *** |
| OTU_2303 1.000 0.000253 *** |
| OTU_1537 1.000 0.000253 *** |
| OTU_736 1.000 0.000253 *** |
| OTU_6217 1.000 0.000253 *** |
| OTU_742 1.000 0.000253 *** |
| OTU_740 1.000 0.000253 *** |
| OTU_2937 1.000 0.000253 *** |
| OTU_638 1.000 0.000253 *** |
| OTU_1687 1.000 0.000253 *** |
| OTU_10477 1.000 0.000253 *** |
| OTU_1358 1.000 0.000253 *** |
| OTU_1781 1.000 0.000253 *** |
| OTU_1641 1.000 0.000253 *** |
| OTU_2319 1.000 0.000253 *** |
| OTU_568 1.000 0.000253 *** |
| OTU_6240 1.000 0.000253 *** |
| OTU_1322 1.000 0.000253 *** |
| OTU_2893 1.000 0.000253 *** |
| OTU_1685 1.000 0.000253 *** |
| OTU_12084 1.000 0.000253 *** |
| OTU_776 1.000 0.000253 *** |
| OTU_2085 1.000 0.000253 *** |
| OTU_1401 1.000 0.000253 *** |
| OTU_605 1.000 0.000253 *** |
| OTU_1130 1.000 0.000253 *** |
| OTU_5219 1.000 0.000253 *** |
| OTU_2867 1.000 0.000253 *** |
| OTU_3476 1.000 0.000253 *** |
| OTU_2230 1.000 0.000253 *** |
| OTU_1479 1.000 0.000253 *** |
| OTU_2306 1.000 0.000253 *** |
| OTU_2376 1.000 0.000253 *** |
| OTU_6848 1.000 0.000253 *** |
| OTU_1298 1.000 0.000253 *** |
| OTU_1158 1.000 0.000253 *** |
| OTU_1363 1.000 0.000253 *** |
| OTU_3399 1.000 0.000253 *** |
| OTU_1766 1.000 0.000253 *** |
| OTU_5166 1.000 0.000253 *** |
| OTU_5205 1.000 0.000253 *** |
| OTU_2344 1.000 0.000253 *** |
| OTU_2139 1.000 0.000253 *** |
| OTU_4707 1.000 0.000253 *** |
| OTU_1145 1.000 0.000253 *** |
| OTU_5710 1.000 0.000253 *** |
| OTU_3003 1.000 0.000253 *** |
| OTU_897 1.000 0.000253 *** |
| OTU_6595 1.000 0.000253 *** |
| OTU_9284 1.000 0.000253 *** |
| OTU_1944 1.000 0.000253 *** |
| OTU_1238 1.000 0.000253 *** |
| OTU_1178 1.000 0.000253 *** |
| OTU_5344 1.000 0.000253 *** |
| OTU_7420 1.000 0.000253 *** |
| OTU_2503 1.000 0.000253 *** |
| OTU_3224 1.000 0.000253 *** |
| OTU_2802 1.000 0.000253 *** |
| OTU_817 1.000 0.000253 *** |
| OTU_3777 1.000 0.000253 *** |
| OTU_11692 1.000 0.000253 *** |
| OTU_7516 1.000 0.000253 *** |
| OTU_1344 1.000 0.000253 *** |
| OTU_5884 1.000 0.000253 *** |
| OTU_1463 1.000 0.000253 *** |
| OTU_1388 1.000 0.000253 *** |
| OTU_3842 1.000 0.000253 *** |
| OTU_8460 1.000 0.000253 *** |
| OTU_1406 1.000 0.000253 *** |
| OTU_3946 1.000 0.000253 *** |
| OTU_4076 1.000 0.000253 *** |
| OTU_3153 1.000 0.000253 *** |
| OTU_1799 1.000 0.000253 *** |
| OTU_3022 1.000 0.000253 *** |
| OTU_2277 1.000 0.000253 *** |
| OTU_1654 1.000 0.000253 *** |
| OTU_6491 1.000 0.000253 *** |
| OTU_1867 1.000 0.000253 *** |
| OTU_2658 1.000 0.000253 *** |
| OTU_1446 1.000 0.000253 *** |
| OTU_2572 1.000 0.000253 *** |
| OTU_2130 1.000 0.000253 *** |
| OTU_1921 1.000 0.000253 *** |
| OTU_2081 1.000 0.000253 *** |
| OTU_1174 1.000 0.000253 *** |
| OTU_887 1.000 0.000253 *** |
| OTU_1546 1.000 0.000253 *** |
| OTU_965 1.000 0.000253 *** |
| OTU_4738 1.000 0.000253 *** |
| OTU_4283 1.000 0.000253 *** |
| OTU_1374 1.000 0.000253 *** |
| OTU_1814 1.000 0.000253 *** |
| OTU_10636 1.000 0.000253 *** |
| OTU_4422 1.000 0.000253 *** |
| OTU_4634 1.000 0.000253 *** |
| OTU_911 1.000 0.000253 *** |
| OTU_3580 1.000 0.000253 *** |
| OTU_5287 1.000 0.000253 *** |
| OTU_2178 1.000 0.000253 *** |
| OTU_9401 1.000 0.000253 *** |
| OTU_979 1.000 0.000253 *** |
| OTU_4992 1.000 0.000253 *** |
| OTU_7339 1.000 0.000253 *** |
| OTU_1076 1.000 0.000253 *** |
| OTU_2977 1.000 0.000253 *** |
| OTU_1670 1.000 0.000253 *** |
| OTU_5068 1.000 0.000253 *** |
| OTU_829 1.000 0.000253 *** |
| OTU_2074 1.000 0.000253 *** |
| OTU_1466 1.000 0.000253 *** |
| OTU_2020 1.000 0.000253 *** |
| OTU_3097 1.000 0.000253 *** |
| OTU_10209 1.000 0.000253 *** |
| OTU_2890 1.000 0.000253 *** |
| OTU_2661 1.000 0.000253 *** |
| OTU_1663 1.000 0.000253 *** |
| OTU_8579 1.000 0.000253 *** |
| OTU_2237 1.000 0.000253 *** |
| OTU_1173 1.000 0.000253 *** |
| OTU_3229 1.000 0.000253 *** |
| OTU_1538 1.000 0.000253 *** |
| OTU_1214 1.000 0.000253 *** |
| OTU_1312 1.000 0.000253 *** |
| OTU_1870 1.000 0.000253 *** |
| OTU_1440 1.000 0.000253 *** |
| OTU_2290 1.000 0.000253 *** |
| OTU_8688 1.000 0.000253 *** |
| OTU_1108 1.000 0.000253 *** |
| OTU_1046 1.000 0.000253 *** |
| OTU_1152 1.000 0.000253 *** |
| OTU_2363 1.000 0.000253 *** |
| OTU_3666 1.000 0.000253 *** |
| OTU_2558 1.000 0.000253 *** |
| OTU_5630 1.000 0.000253 *** |
| OTU_3023 1.000 0.000253 *** |
| OTU_1864 1.000 0.000253 *** |
| OTU_2226 1.000 0.000253 *** |
| OTU_1964 1.000 0.000253 *** |
| OTU_2926 1.000 0.000253 *** |
| OTU_8139 1.000 0.000253 *** |
| OTU_3596 1.000 0.000253 *** |
| OTU_2950 1.000 0.000253 *** |
| OTU_900 1.000 0.000253 *** |
| OTU_1404 1.000 0.000253 *** |
| OTU_1960 1.000 0.000253 *** |
| OTU_4232 1.000 0.000253 *** |
| OTU_2624 1.000 0.000253 *** |
| OTU_4483 1.000 0.000253 *** |
| OTU_1902 1.000 0.000253 *** |
| OTU_11123 1.000 0.000253 *** |
| OTU_1844 1.000 0.000253 *** |
| OTU_3644 1.000 0.000253 *** |
| OTU_7018 1.000 0.000253 *** |
| OTU_3815 1.000 0.000253 *** |
| OTU_3696 1.000 0.000253 *** |
| OTU_2803 1.000 0.000253 *** |
| OTU_1813 1.000 0.000253 *** |
| OTU_2510 1.000 0.000253 *** |
| OTU_2079 1.000 0.000253 *** |
| OTU_1480 1.000 0.000253 *** |
| OTU_1909 1.000 0.000253 *** |
| OTU_2332 1.000 0.000253 *** |
| OTU_1632 1.000 0.000253 *** |
| OTU_2177 1.000 0.000253 *** |
| OTU_3897 1.000 0.000253 *** |
| OTU_1601 1.000 0.000253 *** |
| OTU_2927 1.000 0.000253 *** |
| OTU_2742 1.000 0.000253 *** |
| OTU_2345 1.000 0.000253 *** |
| OTU_2895 1.000 0.000253 *** |
| OTU_3319 1.000 0.000253 *** |
| OTU_3626 1.000 0.000253 *** |
| OTU_1878 1.000 0.000253 *** |
| OTU_4411 1.000 0.000253 *** |
| OTU_3955 1.000 0.000253 *** |
| OTU_1544 1.000 0.000253 *** |
| OTU_1596 1.000 0.000253 *** |
| OTU_1892 1.000 0.000253 *** |
| OTU_10296 1.000 0.000253 *** |
| OTU_2049 1.000 0.000253 *** |
| OTU_3538 1.000 0.000253 *** |
| OTU_1217 1.000 0.000253 *** |
| OTU_2162 1.000 0.000253 *** |
| OTU_2252 1.000 0.000253 *** |
| OTU_2428 1.000 0.000253 *** |
| OTU_4748 1.000 0.000253 *** |
| OTU_4786 1.000 0.000253 *** |
| OTU_2321 1.000 0.000253 *** |
| OTU_3200 1.000 0.000253 *** |
| OTU_4586 1.000 0.000253 *** |
| OTU_2242 1.000 0.000253 *** |
| OTU_7872 1.000 0.000253 *** |
| OTU_3586 1.000 0.000253 *** |
| OTU_6926 1.000 0.000253 *** |
| OTU_1708 1.000 0.000253 *** |
| OTU_10034 1.000 0.000253 *** |
| OTU_2473 1.000 0.000253 *** |
| OTU_3187 1.000 0.000253 *** |
| OTU_1811 1.000 0.000253 *** |
| OTU_8831 1.000 0.000253 *** |
| OTU_3529 1.000 0.000253 *** |
| OTU_2056 1.000 0.000253 *** |
| OTU_5526 1.000 0.000253 *** |
| OTU_2570 1.000 0.000253 *** |
| OTU_7456 1.000 0.000253 *** |
| OTU_2781 1.000 0.000253 *** |
| OTU_5857 1.000 0.000253 *** |
| OTU_4364 1.000 0.000253 *** |
| OTU_7065 1.000 0.000253 *** |
| OTU_2346 1.000 0.000253 *** |
| OTU_3723 1.000 0.000253 *** |
| OTU_2164 1.000 0.000253 *** |
| OTU_9837 1.000 0.000253 *** |
| OTU_1869 1.000 0.000253 *** |
| OTU_1829 1.000 0.000253 *** |
| OTU_5763 1.000 0.000253 *** |
| OTU_2343 1.000 0.000253 *** |
| OTU_2682 1.000 0.000253 *** |
| OTU_2791 1.000 0.000253 *** |
| OTU_2377 1.000 0.000253 *** |
| OTU_3087 1.000 0.000253 *** |
| OTU_3768 1.000 0.000253 *** |
| OTU_4442 1.000 0.000253 *** |
| OTU_3214 1.000 0.000253 *** |
| OTU_3383 1.000 0.000253 *** |
| OTU_2744 1.000 0.000253 *** |
| OTU_1764 1.000 0.000253 *** |
| OTU_2125 1.000 0.000253 *** |
| OTU_2755 1.000 0.000253 *** |
| OTU_2269 1.000 0.000253 *** |
| OTU_2325 1.000 0.000253 *** |
| OTU_4242 1.000 0.000253 *** |
| OTU_1997 1.000 0.000253 *** |
| OTU_4308 1.000 0.000253 *** |
| OTU_11439 1.000 0.000253 *** |
| OTU_3234 1.000 0.000253 *** |
| OTU_3713 1.000 0.000253 *** |
| OTU_6846 1.000 0.000253 *** |
| OTU_7379 1.000 0.000253 *** |
| OTU_3582 1.000 0.000253 *** |
| OTU_2604 1.000 0.000253 *** |
| OTU_7260 1.000 0.000253 *** |
| OTU_4794 1.000 0.000253 *** |
| OTU_2933 1.000 0.000253 *** |
| OTU_3060 1.000 0.000253 *** |
| OTU_2258 1.000 0.000253 *** |
| OTU_2151 1.000 0.000253 *** |
| OTU_2981 1.000 0.000253 *** |
| OTU_1602 1.000 0.000253 *** |
| OTU_8934 1.000 0.000253 *** |
| OTU_4326 1.000 0.000253 *** |
| OTU_5254 1.000 0.000253 *** |
| OTU_1879 1.000 0.000253 *** |
| OTU_4525 1.000 0.000253 *** |
| OTU_3368 1.000 0.000253 *** |
| OTU_2274 1.000 0.000253 *** |
| OTU_2825 1.000 0.000253 *** |
| OTU_3602 1.000 0.000253 *** |
| OTU_5016 1.000 0.000253 *** |
| OTU_3328 1.000 0.000253 *** |
| OTU_5280 1.000 0.000253 *** |
| OTU_3764 1.000 0.000253 *** |
| OTU_4404 1.000 0.000253 *** |
| OTU_10752 1.000 0.000253 *** |
| OTU_262 1.000 0.000253 *** |
| OTU_297 1.000 0.000253 *** |
| OTU_225 1.000 0.000253 *** |
| OTU_313 1.000 0.000253 *** |
| OTU_108 1.000 0.000253 *** |
| OTU_141 1.000 0.000253 *** |
| OTU_159 1.000 0.000253 *** |
| OTU_456 1.000 0.000253 *** |
| OTU_181 1.000 0.000253 *** |
| OTU_584 1.000 0.000253 *** |
| OTU_395 1.000 0.000253 *** |
| OTU_437 1.000 0.000253 *** |
| OTU_552 1.000 0.000253 *** |
| OTU_1904 1.000 0.000253 *** |
| OTU_1111 1.000 0.000253 *** |
| OTU_545 1.000 0.000253 *** |
| OTU_298 1.000 0.000253 *** |
| OTU_789 1.000 0.000253 *** |
| OTU_384 1.000 0.000253 *** |
| OTU_1637 1.000 0.000253 *** |
| OTU_1584 1.000 0.000253 *** |
| OTU_503 1.000 0.000253 *** |
| OTU_527 1.000 0.000253 *** |
| OTU_547 1.000 0.000253 *** |
| OTU_856 1.000 0.000253 *** |
| OTU_228 1.000 0.000253 *** |
| OTU_505 1.000 0.000253 *** |
| OTU_635 1.000 0.000253 *** |
| OTU_613 0.999 0.000253 *** |
| OTU_575 0.999 0.000253 *** |
| OTU_888 0.999 0.000253 *** |
| OTU_431 0.999 0.000253 *** |
| OTU_6324 0.999 0.000253 *** |
| OTU_3248 0.999 0.000253 *** |
| OTU_630 0.999 0.000253 *** |
| OTU_1318 0.999 0.000253 *** |
| OTU_232 0.999 0.000253 *** |
| OTU_490 0.999 0.000253 *** |
| OTU_162 0.999 0.000253 *** |
| OTU_487 0.999 0.000253 *** |
| OTU_760 0.999 0.000253 *** |
| OTU_3712 0.999 0.000253 *** |
| OTU_449 0.999 0.000253 *** |
| OTU_665 0.999 0.000253 *** |
| OTU_9920 0.999 0.000253 *** |
| OTU_202 0.999 0.000253 *** |
| OTU_589 0.999 0.000253 *** |
| OTU_1095 0.999 0.000253 *** |
| OTU_1006 0.999 0.000253 *** |
| OTU_854 0.999 0.000253 *** |
| OTU_1278 0.999 0.000253 *** |
| OTU_84 0.999 0.000253 *** |
| OTU_803 0.999 0.000253 *** |
| OTU_846 0.999 0.000253 *** |
| OTU_193 0.999 0.000253 *** |
| OTU_55 0.999 0.000253 *** |
| OTU_567 0.999 0.000253 *** |
| OTU_428 0.999 0.000253 *** |
| OTU_10193 0.999 0.000253 *** |
| OTU_10 0.999 0.000253 *** |
| OTU_1109 0.999 0.000253 *** |
| OTU_483 0.999 0.000253 *** |
| OTU_1043 0.999 0.000253 *** |
| OTU_107 0.999 0.000253 *** |
| OTU_1683 0.999 0.000253 *** |
| OTU_1622 0.999 0.000253 *** |
| OTU_1134 0.999 0.000253 *** |
| OTU_257 0.999 0.000253 *** |
| OTU_1496 0.999 0.000253 *** |
| OTU_1986 0.999 0.000253 *** |
| OTU_8090 0.999 0.000253 *** |
| OTU_1232 0.999 0.000253 *** |
| OTU_946 0.999 0.000253 *** |
| OTU_1338 0.999 0.000253 *** |
| OTU_342 0.999 0.000253 *** |
| OTU_1304 0.999 0.000253 *** |
| OTU_659 0.999 0.000253 *** |
| OTU_315 0.999 0.000253 *** |
| OTU_705 0.999 0.000253 *** |
| OTU_459 0.999 0.000253 *** |
| OTU_484 0.999 0.000253 *** |
| OTU_3400 0.999 0.000253 *** |
| OTU_1593 0.999 0.000253 *** |
| OTU_872 0.999 0.000253 *** |
| OTU_4004 0.999 0.000253 *** |
| OTU_1007 0.999 0.000253 *** |
| OTU_988 0.999 0.000253 *** |
| OTU_155 0.999 0.000253 *** |
| OTU_842 0.999 0.000253 *** |
| OTU_1853 0.999 0.000253 *** |
| OTU_311 0.999 0.000253 *** |
| OTU_393 0.999 0.000253 *** |
| OTU_3295 0.999 0.000253 *** |
| OTU_1487 0.999 0.000253 *** |
| OTU_205 0.999 0.000253 *** |
| OTU_1053 0.999 0.000253 *** |
| OTU_1625 0.999 0.000253 *** |
| OTU_868 0.999 0.000253 *** |
| OTU_2901 0.999 0.000253 *** |
| OTU_430 0.999 0.000253 *** |
| OTU_8876 0.999 0.000253 *** |
| OTU_1120 0.999 0.000253 *** |
| OTU_464 0.999 0.000253 *** |
| OTU_1691 0.999 0.000253 *** |
| OTU_11206 0.999 0.000253 *** |
| OTU_1980 0.999 0.000253 *** |
| OTU_1389 0.999 0.000253 *** |
| OTU_1036 0.998 0.000253 *** |
| OTU_280 0.998 0.000253 *** |
| OTU_940 0.998 0.000253 *** |
| OTU_1861 0.998 0.000253 *** |
| OTU_1560 0.998 0.000253 *** |
| OTU_1483 0.998 0.000253 *** |
| OTU_758 0.998 0.000253 *** |
| OTU_12185 0.998 0.000253 *** |
| OTU_196 0.998 0.000253 *** |
| OTU_143 0.998 0.000253 *** |
| OTU_1918 0.998 0.000253 *** |
| OTU_10239 0.998 0.000253 *** |
| OTU_2209 0.998 0.000253 *** |
| OTU_1295 0.998 0.000253 *** |
| OTU_2808 0.998 0.000253 *** |
| OTU_1432 0.998 0.000253 *** |
| OTU_1399 0.998 0.000253 *** |
| OTU_1103 0.998 0.000253 *** |
| OTU_2050 0.998 0.000253 *** |
| OTU_2236 0.998 0.000253 *** |
| OTU_1198 0.998 0.000253 *** |
| OTU_489 0.998 0.000253 *** |
| OTU_1063 0.998 0.000253 *** |
| OTU_1549 0.998 0.000253 *** |
| OTU_481 0.998 0.000253 *** |
| OTU_1916 0.998 0.000253 *** |
| OTU_1019 0.998 0.000253 *** |
| OTU_3045 0.998 0.000253 *** |
| OTU_192 0.998 0.000253 *** |
| OTU_4251 0.998 0.000253 *** |
| OTU_266 0.998 0.000253 *** |
| OTU_2435 0.998 0.000253 *** |
| OTU_2045 0.998 0.000253 *** |
| OTU_258 0.998 0.000253 *** |
| OTU_2263 0.998 0.000253 *** |
| OTU_1613 0.998 0.000253 *** |
| OTU_1717 0.998 0.000253 *** |
| OTU_1261 0.998 0.000253 *** |
| OTU_1383 0.998 0.000253 *** |
| OTU_845 0.998 0.000253 *** |
| OTU_366 0.998 0.000253 *** |
| OTU_1236 0.998 0.000253 *** |
| OTU_365 0.998 0.000253 *** |
| OTU_52 0.998 0.000253 *** |
| OTU_622 0.998 0.000253 *** |
| OTU_165 0.998 0.000253 *** |
| OTU_1269 0.998 0.000253 *** |
| OTU_689 0.998 0.000253 *** |
| OTU_12103 0.998 0.000253 *** |
| OTU_1376 0.998 0.000253 *** |
| OTU_2587 0.998 0.000253 *** |
| OTU_189 0.998 0.000253 *** |
| OTU_2556 0.998 0.000253 *** |
| OTU_1197 0.998 0.000253 *** |
| OTU_271 0.998 0.000253 *** |
| OTU_966 0.998 0.000253 *** |
| OTU_801 0.998 0.000253 *** |
| OTU_11764 0.998 0.000253 *** |
| OTU_4595 0.998 0.000253 *** |
| OTU_1494 0.998 0.000253 *** |
| OTU_2387 0.998 0.000253 *** |
| OTU_1495 0.998 0.000253 *** |
| OTU_2025 0.998 0.000253 *** |
| OTU_2493 0.998 0.000253 *** |
| OTU_11081 0.998 0.000253 *** |
| OTU_579 0.998 0.000253 *** |
| OTU_517 0.998 0.000253 *** |
| OTU_1803 0.998 0.000253 *** |
| OTU_1384 0.998 0.000253 *** |
| OTU_1179 0.998 0.000253 *** |
| OTU_1785 0.998 0.000253 *** |
| OTU_1658 0.998 0.000253 *** |
| OTU_1449 0.998 0.000253 *** |
| OTU_82 0.998 0.000253 *** |
| OTU_1727 0.998 0.000253 *** |
| OTU_694 0.998 0.000253 *** |
| OTU_9931 0.997 0.000253 *** |
| OTU_7691 0.997 0.000253 *** |
| OTU_1849 0.997 0.000253 *** |
| OTU_1114 0.997 0.000253 *** |
| OTU_239 0.997 0.000253 *** |
| OTU_6235 0.997 0.000253 *** |
| OTU_623 0.997 0.000253 *** |
| OTU_3975 0.997 0.000253 *** |
| OTU_305 0.997 0.000253 *** |
| OTU_714 0.997 0.000253 *** |
| OTU_1430 0.997 0.000253 *** |
| OTU_1462 0.997 0.000253 *** |
| OTU_1370 0.997 0.000253 *** |
| OTU_1590 0.997 0.000253 *** |
| OTU_570 0.997 0.000253 *** |
| OTU_1905 0.997 0.000253 *** |
| OTU_418 0.997 0.000253 *** |
| OTU_912 0.997 0.000253 *** |
| OTU_909 0.997 0.000253 *** |
| OTU_1735 0.997 0.000253 *** |
| OTU_1443 0.997 0.000253 *** |
| OTU_5836 0.997 0.000253 *** |
| OTU_1289 0.997 0.000253 *** |
| OTU_493 0.997 0.000253 *** |
| OTU_3561 0.997 0.000253 *** |
| OTU_1566 0.997 0.000253 *** |
| OTU_992 0.997 0.000253 *** |
| OTU_3150 0.997 0.000253 *** |
| OTU_1452 0.997 0.000253 *** |
| OTU_1055 0.997 0.000253 *** |
| OTU_2484 0.997 0.000253 *** |
| OTU_4418 0.997 0.000253 *** |
| OTU_7799 0.997 0.000253 *** |
| OTU_1787 0.997 0.000253 *** |
| OTU_690 0.997 0.000253 *** |
| OTU_3969 0.997 0.000253 *** |
| OTU_1273 0.997 0.000253 *** |
| OTU_240 0.997 0.000253 *** |
| OTU_805 0.997 0.000253 *** |
| OTU_337 0.997 0.000253 *** |
| OTU_2033 0.997 0.000253 *** |
| OTU_774 0.997 0.000253 *** |
| OTU_2688 0.997 0.000253 *** |
| OTU_1877 0.997 0.000253 *** |
| OTU_3523 0.997 0.000253 *** |
| OTU_3566 0.997 0.000253 *** |
| OTU_2520 0.997 0.000253 *** |
| OTU_11788 0.997 0.000253 *** |
| OTU_1254 0.997 0.000253 *** |
| OTU_697 0.997 0.000253 *** |
| OTU_2279 0.997 0.000253 *** |
| OTU_877 0.997 0.000253 *** |
| OTU_2191 0.997 0.000253 *** |
| OTU_3688 0.997 0.000253 *** |
| OTU_1299 0.997 0.000253 *** |
| OTU_1138 0.997 0.000253 *** |
| OTU_1142 0.997 0.000253 *** |
| OTU_2886 0.997 0.000253 *** |
| OTU_93 0.997 0.000253 *** |
| OTU_217 0.997 0.000253 *** |
| OTU_2444 0.997 0.000253 *** |
| OTU_2880 0.997 0.000253 *** |
| OTU_728 0.997 0.000253 *** |
| OTU_1146 0.996 0.000253 *** |
| OTU_1828 0.996 0.000253 *** |
| OTU_2827 0.996 0.000253 *** |
| OTU_748 0.996 0.000253 *** |
| OTU_989 0.996 0.000253 *** |
| OTU_2394 0.996 0.000253 *** |
| OTU_7888 0.996 0.000253 *** |
| OTU_3550 0.996 0.000253 *** |
| OTU_1843 0.996 0.000253 *** |
| OTU_2638 0.996 0.000253 *** |
| OTU_268 0.996 0.000253 *** |
| OTU_1901 0.996 0.000253 *** |
| OTU_7302 0.996 0.000253 *** |
| OTU_67 0.996 0.000253 *** |
| OTU_6094 0.996 0.000253 *** |
| OTU_10065 0.996 0.000253 *** |
| OTU_2523 0.996 0.000253 *** |
| OTU_1252 0.996 0.000253 *** |
| OTU_358 0.996 0.000253 *** |
| OTU_1407 0.996 0.000253 *** |
| OTU_1256 0.996 0.000253 *** |
| OTU_1163 0.996 0.000253 *** |
| OTU_410 0.996 0.000253 *** |
| OTU_283 0.996 0.000253 *** |
| OTU_1783 0.996 0.000253 *** |
| OTU_316 0.996 0.000253 *** |
| OTU_444 0.996 0.000253 *** |
| OTU_9785 0.996 0.000253 *** |
| OTU_1655 0.996 0.000253 *** |
| OTU_976 0.996 0.000253 *** |
| OTU_1202 0.996 0.000253 *** |
| OTU_1270 0.996 0.000253 *** |
| OTU_1563 0.996 0.000253 *** |
| OTU_1119 0.996 0.000253 *** |
| OTU_1737 0.996 0.000253 *** |
| OTU_3040 0.996 0.000253 *** |
| OTU_11897 0.996 0.000253 *** |
| OTU_8116 0.996 0.000253 *** |
| OTU_486 0.996 0.000253 *** |
| OTU_858 0.996 0.000253 *** |
| OTU_1931 0.996 0.000253 *** |
| OTU_1678 0.996 0.000253 *** |
| OTU_1485 0.996 0.000253 *** |
| OTU_1580 0.996 0.000253 *** |
| OTU_1033 0.996 0.000253 *** |
| OTU_739 0.996 0.000253 *** |
| OTU_3302 0.996 0.000253 *** |
| OTU_1184 0.996 0.000253 *** |
| OTU_10168 0.996 0.000253 *** |
| OTU_810 0.996 0.000253 *** |
| OTU_3545 0.995 0.000253 *** |
| OTU_1048 0.995 0.000253 *** |
| OTU_5353 0.995 0.000253 *** |
| OTU_555 0.995 0.000253 *** |
| OTU_10282 0.995 0.000253 *** |
| OTU_2487 0.995 0.000253 *** |
| OTU_1484 0.995 0.000253 *** |
| OTU_1366 0.995 0.000253 *** |
| OTU_114 0.995 0.000253 *** |
| OTU_497 0.995 0.000253 *** |
| OTU_745 0.995 0.000253 *** |
| OTU_213 0.995 0.000253 *** |
| OTU_768 0.995 0.000253 *** |
| OTU_4548 0.995 0.000253 *** |
| OTU_761 0.995 0.000253 *** |
| OTU_4434 0.995 0.000253 *** |
| OTU_317 0.995 0.000253 *** |
| OTU_3115 0.995 0.000253 *** |
| OTU_971 0.995 0.000253 *** |
| OTU_677 0.995 0.000253 *** |
| OTU_731 0.995 0.000253 *** |
| OTU_1195 0.995 0.000253 *** |
| OTU_850 0.995 0.000253 *** |
| OTU_73 0.995 0.000253 *** |
| OTU_3174 0.995 0.000253 *** |
| OTU_1745 0.995 0.000253 *** |
| OTU_3395 0.995 0.000253 *** |
| OTU_2858 0.995 0.000253 *** |
| OTU_4957 0.995 0.000253 *** |
| OTU_639 0.995 0.000253 *** |
| OTU_681 0.995 0.000253 *** |
| OTU_1021 0.995 0.000253 *** |
| OTU_2107 0.995 0.000253 *** |
| OTU_2873 0.995 0.000253 *** |
| OTU_1010 0.995 0.000253 *** |
| OTU_825 0.995 0.000253 *** |
| OTU_356 0.995 0.000253 *** |
| OTU_1170 0.995 0.000253 *** |
| OTU_2251 0.995 0.000253 *** |
| OTU_2001 0.995 0.000253 *** |
| OTU_2902 0.995 0.000253 *** |
| OTU_1937 0.995 0.000253 *** |
| OTU_2156 0.995 0.000253 *** |
| OTU_7793 0.995 0.000253 *** |
| OTU_1431 0.995 0.000253 *** |
| OTU_773 0.995 0.000253 *** |
| OTU_1263 0.995 0.000253 *** |
| OTU_1606 0.995 0.000253 *** |
| OTU_831 0.995 0.000253 *** |
| OTU_4845 0.995 0.000253 *** |
| OTU_1720 0.995 0.000253 *** |
| OTU_5438 0.995 0.000253 *** |
| OTU_1325 0.995 0.000253 *** |
| OTU_3354 0.994 0.000253 *** |
| OTU_1749 0.994 0.000253 *** |
| OTU_2142 0.994 0.000253 *** |
| OTU_1199 0.994 0.000253 *** |
| OTU_1472 0.994 0.000253 *** |
| OTU_8566 0.994 0.000253 *** |
| OTU_4690 0.994 0.000253 *** |
| OTU_2223 0.994 0.000253 *** |
| OTU_886 0.994 0.000253 *** |
| OTU_1981 0.994 0.000253 *** |
| OTU_1054 0.994 0.000253 *** |
| OTU_7667 0.994 0.000253 *** |
| OTU_2054 0.994 0.000253 *** |
| OTU_500 0.994 0.000253 *** |
| OTU_2034 0.994 0.000253 *** |
| OTU_267 0.994 0.000253 *** |
| OTU_2768 0.994 0.000253 *** |
| OTU_593 0.994 0.000253 *** |
| OTU_1456 0.994 0.000253 *** |
| OTU_1806 0.994 0.000253 *** |
| OTU_981 0.994 0.000253 *** |
| OTU_673 0.994 0.000253 *** |
| OTU_54 0.994 0.000253 *** |
| OTU_3816 0.994 0.000253 *** |
| OTU_1004 0.994 0.000253 *** |
| OTU_1930 0.994 0.000253 *** |
| OTU_1473 0.994 0.000253 *** |
| OTU_36 0.994 0.000253 *** |
| OTU_985 0.994 0.000253 *** |
| OTU_381 0.994 0.000253 *** |
| OTU_8596 0.994 0.000253 *** |
| OTU_1628 0.994 0.000253 *** |
| OTU_3140 0.994 0.000253 *** |
| OTU_3710 0.994 0.000253 *** |
| OTU_1872 0.994 0.000253 *** |
| OTU_2161 0.994 0.000253 *** |
| OTU_1275 0.994 0.000253 *** |
| OTU_5133 0.994 0.000253 *** |
| OTU_303 0.994 0.000253 *** |
| OTU_2121 0.994 0.000253 *** |
| OTU_167 0.994 0.000253 *** |
| OTU_1489 0.994 0.000253 *** |
| OTU_1365 0.994 0.000253 *** |
| OTU_1160 0.994 0.000253 *** |
| OTU_1267 0.994 0.000253 *** |
| OTU_2096 0.994 0.000253 *** |
| OTU_140 0.994 0.000253 *** |
| OTU_1681 0.994 0.000253 *** |
| OTU_625 0.994 0.000253 *** |
| OTU_338 0.994 0.000253 *** |
| OTU_1074 0.994 0.000253 *** |
| OTU_328 0.993 0.000253 *** |
| OTU_208 0.993 0.000253 *** |
| OTU_893 0.993 0.000253 *** |
| OTU_3018 0.993 0.000253 *** |
| OTU_3535 0.993 0.000253 *** |
| OTU_132 0.993 0.000253 *** |
| OTU_962 0.993 0.000253 *** |
| OTU_3233 0.993 0.000253 *** |
| OTU_368 0.993 0.000253 *** |
| OTU_1467 0.993 0.000253 *** |
| OTU_521 0.993 0.000253 *** |
| OTU_8454 0.993 0.000253 *** |
| OTU_4816 0.993 0.000253 *** |
| OTU_2884 0.993 0.000253 *** |
| OTU_1094 0.993 0.000253 *** |
| OTU_2199 0.993 0.000253 *** |
| OTU_2488 0.993 0.000253 *** |
| OTU_3056 0.993 0.000253 *** |
| OTU_203 0.993 0.000253 *** |
| OTU_765 0.993 0.000253 *** |
| OTU_241 0.993 0.000253 *** |
| OTU_461 0.993 0.000253 *** |
| OTU_963 0.993 0.000253 *** |
| OTU_969 0.993 0.000253 *** |
| OTU_944 0.993 0.000253 *** |
| OTU_4413 0.993 0.000253 *** |
| OTU_6181 0.992 0.000253 *** |
| OTU_706 0.992 0.000253 *** |
| OTU_680 0.992 0.000253 *** |
| OTU_907 0.992 0.000253 *** |
| OTU_627 0.992 0.000253 *** |
| OTU_3994 0.992 0.000253 *** |
| OTU_1835 0.992 0.000253 *** |
| OTU_2470 0.992 0.000253 *** |
| OTU_446 0.992 0.000253 *** |
| OTU_640 0.992 0.000253 *** |
| OTU_1066 0.992 0.000253 *** |
| OTU_2432 0.992 0.000253 *** |
| OTU_1635 0.992 0.000253 *** |
| OTU_1833 0.992 0.000253 *** |
| OTU_7062 0.992 0.000253 *** |
| OTU_1719 0.992 0.000253 *** |
| OTU_263 0.992 0.000253 *** |
| OTU_1917 0.992 0.000253 *** |
| OTU_642 0.992 0.000253 *** |
| OTU_1477 0.992 0.000253 *** |
| OTU_3349 0.992 0.000253 *** |
| OTU_1609 0.992 0.000253 *** |
| OTU_1615 0.992 0.000253 *** |
| OTU_2110 0.992 0.000253 *** |
| OTU_2122 0.991 0.000253 *** |
| OTU_1315 0.991 0.000253 *** |
| OTU_1122 0.991 0.000253 *** |
| OTU_2405 0.991 0.000253 *** |
| OTU_1786 0.991 0.000253 *** |
| OTU_3888 0.991 0.000253 *** |
| OTU_139 0.991 0.000253 *** |
| OTU_286 0.991 0.000253 *** |
| OTU_781 0.991 0.000253 *** |
| OTU_9452 0.991 0.000253 *** |
| OTU_4016 0.991 0.000253 *** |
| OTU_1574 0.991 0.000253 *** |
| OTU_2358 0.991 0.000253 *** |
| OTU_3235 0.991 0.000253 *** |
| OTU_4620 0.991 0.000253 *** |
| OTU_1548 0.991 0.000253 *** |
| OTU_2355 0.991 0.000253 *** |
| OTU_2887 0.991 0.000253 *** |
| OTU_7855 0.991 0.000253 *** |
| OTU_1510 0.991 0.000253 *** |
| OTU_23 0.990 0.000253 *** |
| OTU_1608 0.990 0.000253 *** |
| OTU_10839 0.990 0.000253 *** |
| OTU_3603 0.990 0.000253 *** |
| OTU_1696 0.990 0.000253 *** |
| OTU_248 0.990 0.000253 *** |
| OTU_523 0.990 0.000253 *** |
| OTU_5924 0.990 0.000253 *** |
| OTU_3431 0.990 0.000253 *** |
| OTU_5414 0.990 0.000253 *** |
| OTU_8817 0.990 0.000253 *** |
| OTU_8028 0.990 0.000253 *** |
| OTU_2350 0.990 0.000253 *** |
| OTU_931 0.990 0.000253 *** |
| OTU_2134 0.990 0.000253 *** |
| OTU_1885 0.990 0.000253 *** |
| OTU_1382 0.989 0.000253 *** |
| OTU_881 0.989 0.000253 *** |
| OTU_9204 0.989 0.000253 *** |
| OTU_2498 0.989 0.000253 *** |
| OTU_2598 0.989 0.000253 *** |
| OTU_1711 0.989 0.000253 *** |
| OTU_1810 0.989 0.000253 *** |
| OTU_1640 0.989 0.000253 *** |
| OTU_2466 0.989 0.000253 *** |
| OTU_522 0.989 0.000253 *** |
| OTU_1099 0.989 0.000253 *** |
| OTU_899 0.989 0.000253 *** |
| OTU_783 0.989 0.000253 *** |
| OTU_695 0.989 0.000253 *** |
| OTU_8305 0.989 0.000253 *** |
| OTU_6637 0.988 0.000253 *** |
| OTU_4635 0.988 0.000253 *** |
| OTU_4356 0.988 0.000253 *** |
| OTU_1293 0.988 0.000253 *** |
| OTU_274 0.988 0.000253 *** |
| OTU_11478 0.988 0.000253 *** |
| OTU_2816 0.988 0.000253 *** |
| OTU_439 0.988 0.000253 *** |
| OTU_2975 0.988 0.000253 *** |
| OTU_1221 0.988 0.000253 *** |
| OTU_1450 0.988 0.000253 *** |
| OTU_1123 0.988 0.000253 *** |
| OTU_289 0.988 0.000253 *** |
| OTU_160 0.988 0.000253 *** |
| OTU_603 0.988 0.000253 *** |
| OTU_10118 0.988 0.000253 *** |
| OTU_1527 0.988 0.000253 *** |
| OTU_2250 0.988 0.000253 *** |
| OTU_1011 0.988 0.000253 *** |
| OTU_3232 0.988 0.000253 *** |
| OTU_178 0.988 0.000253 *** |
| OTU_1564 0.987 0.000253 *** |
| OTU_3304 0.987 0.000253 *** |
| OTU_2260 0.987 0.000253 *** |
| OTU_2494 0.987 0.000253 *** |
| OTU_1514 0.987 0.000253 *** |
| OTU_2454 0.987 0.000253 *** |
| OTU_1791 0.987 0.000253 *** |
| OTU_382 0.987 0.000253 *** |
| OTU_7772 0.987 0.000253 *** |
| OTU_1526 0.987 0.000253 *** |
| OTU_397 0.987 0.000253 *** |
| OTU_804 0.987 0.000253 *** |
| OTU_1309 0.987 0.000253 *** |
| OTU_2784 0.986 0.000253 *** |
| OTU_419 0.986 0.000253 *** |
| OTU_1771 0.986 0.000253 *** |
| OTU_1428 0.986 0.000253 *** |
| OTU_3769 0.986 0.000253 *** |
| OTU_691 0.986 0.000253 *** |
| OTU_3984 0.986 0.000253 *** |
| OTU_1191 0.986 0.000253 *** |
| OTU_2324 0.986 0.000253 *** |
| OTU_46 0.986 0.000253 *** |
| OTU_249 0.986 0.000253 *** |
| OTU_3627 0.986 0.000253 *** |
| OTU_133 0.986 0.000253 *** |
| OTU_2477 0.986 0.000253 *** |
| OTU_1282 0.986 0.000253 *** |
| OTU_1097 0.985 0.000253 *** |
| OTU_4288 0.985 0.000253 *** |
| OTU_4236 0.985 0.000253 *** |
| OTU_901 0.985 0.000253 *** |
| OTU_172 0.985 0.000253 *** |
| OTU_2315 0.985 0.000253 *** |
| OTU_9940 0.985 0.000253 *** |
| OTU_97 0.984 0.000253 *** |
| OTU_10088 0.984 0.000253 *** |
| OTU_7373 0.984 0.000253 *** |
| OTU_1503 0.984 0.000253 *** |
| OTU_670 0.984 0.000253 *** |
| OTU_3148 0.984 0.000253 *** |
| OTU_7575 0.984 0.000253 *** |
| OTU_648 0.984 0.000253 *** |
| OTU_4187 0.984 0.000253 *** |
| OTU_3183 0.984 0.000253 *** |
| OTU_649 0.984 0.000253 *** |
| OTU_510 0.984 0.000253 *** |
| OTU_194 0.983 0.000253 *** |
| OTU_1375 0.983 0.000253 *** |
| OTU_2272 0.983 0.000253 *** |
| OTU_1078 0.983 0.000253 *** |
| OTU_1351 0.983 0.000253 *** |
| OTU_1347 0.983 0.000253 *** |
| OTU_1294 0.983 0.000253 *** |
| OTU_201 0.983 0.000253 *** |
| OTU_322 0.983 0.000253 *** |
| OTU_9445 0.983 0.000253 *** |
| OTU_3933 0.983 0.000253 *** |
| OTU_290 0.983 0.000253 *** |
| OTU_729 0.983 0.000253 *** |
| OTU_5432 0.983 0.000253 *** |
| OTU_88 0.983 0.000253 *** |
| OTU_221 0.983 0.000253 *** |
| OTU_1620 0.982 0.000253 *** |
| OTU_1712 0.982 0.000253 *** |
| OTU_443 0.982 0.000253 *** |
| OTU_5636 0.982 0.000253 *** |
| OTU_2184 0.982 0.000253 *** |
| OTU_1644 0.981 0.000253 *** |
| OTU_278 0.981 0.000253 *** |
| OTU_5105 0.981 0.000253 *** |
| OTU_1439 0.981 0.000253 *** |
| OTU_1914 0.981 0.000253 *** |
| OTU_10714 0.981 0.000253 *** |
| OTU_7688 0.981 0.000253 *** |
| OTU_730 0.981 0.000253 *** |
| OTU_1161 0.981 0.000253 *** |
| OTU_1020 0.981 0.000253 *** |
| OTU_687 0.981 0.000253 *** |
| OTU_310 0.981 0.000461 *** |
| OTU_269 0.981 0.000253 *** |
| OTU_2421 0.981 0.000253 *** |
| OTU_1058 0.981 0.000253 *** |
| OTU_4467 0.981 0.000253 *** |
| OTU_1941 0.980 0.000253 *** |
| OTU_3998 0.980 0.000253 *** |
| OTU_1039 0.980 0.000253 *** |
| OTU_1131 0.980 0.000253 *** |
| OTU_688 0.979 0.000253 *** |
| OTU_1083 0.979 0.000253 *** |
| OTU_1437 0.979 0.000253 *** |
| OTU_3195 0.978 0.000253 *** |
| OTU_2002 0.978 0.000253 *** |
| OTU_1069 0.978 0.000253 *** |
| OTU_1156 0.978 0.000253 *** |
| OTU_2089 0.978 0.000253 *** |
| OTU_1603 0.978 0.000253 *** |
| OTU_229 0.977 0.000253 *** |
| OTU_2335 0.977 0.000253 *** |
| OTU_29 0.977 0.000253 *** |
| OTU_1767 0.977 0.000253 *** |
| OTU_1144 0.977 0.000253 *** |
| OTU_821 0.976 0.000253 *** |
| OTU_562 0.976 0.000253 *** |
| OTU_571 0.976 0.000253 *** |
| OTU_1975 0.976 0.000253 *** |
| OTU_710 0.976 0.000253 *** |
| OTU_10075 0.976 0.000253 *** |
| OTU_1379 0.976 0.000253 *** |
| OTU_134 0.976 0.000253 *** |
| OTU_2823 0.975 0.000253 *** |
| OTU_5819 0.975 0.000253 *** |
| OTU_7645 0.975 0.000253 *** |
| OTU_1589 0.975 0.000253 *** |
| OTU_1017 0.975 0.000253 *** |
| OTU_12273 0.975 0.000253 *** |
| OTU_3646 0.975 0.000253 *** |
| OTU_3055 0.974 0.000253 *** |
| OTU_1895 0.974 0.000253 *** |
| OTU_6927 0.974 0.000253 *** |
| OTU_3285 0.974 0.000253 *** |
| OTU_10994 0.974 0.000253 *** |
| OTU_3043 0.974 0.000253 *** |
| OTU_5837 0.973 0.000253 *** |
| OTU_8982 0.973 0.000253 *** |
| OTU_11773 0.973 0.000253 *** |
| OTU_1922 0.973 0.000253 *** |
| OTU_1037 0.972 0.000253 *** |
| OTU_1274 0.972 0.000253 *** |
| OTU_770 0.972 0.000253 *** |
| OTU_1684 0.972 0.000253 *** |
| OTU_441 0.971 0.000253 *** |
| OTU_1090 0.971 0.000253 *** |
| OTU_1082 0.971 0.000253 *** |
| OTU_1337 0.971 0.000253 *** |
| OTU_5732 0.971 0.000253 *** |
| OTU_1694 0.971 0.000253 *** |
| OTU_910 0.970 0.000253 *** |
| OTU_585 0.970 0.000253 *** |
| OTU_8528 0.970 0.000253 *** |
| OTU_2997 0.970 0.000253 *** |
| OTU_1414 0.970 0.000253 *** |
| OTU_25 0.969 0.000253 *** |
| OTU_3065 0.969 0.000253 *** |
| OTU_580 0.968 0.000253 *** |
| OTU_631 0.967 0.000253 *** |
| OTU_851 0.967 0.000253 *** |
| OTU_861 0.967 0.000253 *** |
| OTU_838 0.966 0.000253 *** |
| OTU_2611 0.965 0.000253 *** |
| OTU_3209 0.965 0.000253 *** |
| OTU_903 0.964 0.000253 *** |
| OTU_884 0.963 0.000253 *** |
| OTU_2009 0.963 0.000253 *** |
| OTU_216 0.962 0.001931 ** |
| OTU_2132 0.962 0.000253 *** |
| OTU_4327 0.962 0.000253 *** |
| OTU_331 0.962 0.000253 *** |
| OTU_573 0.962 0.000253 *** |
| OTU_1167 0.961 0.000253 *** |
| OTU_3445 0.961 0.000253 *** |
| OTU_513 0.961 0.000253 *** |
| OTU_4233 0.960 0.000253 *** |
| OTU_1041 0.960 0.000253 *** |
| OTU_5067 0.959 0.000253 *** |
| OTU_429 0.959 0.000253 *** |
| OTU_1249 0.958 0.000253 *** |
| OTU_1087 0.958 0.000253 *** |
| OTU_4176 0.957 0.000253 *** |
| OTU_543 0.957 0.000253 *** |
| OTU_2616 0.957 0.000253 *** |
| OTU_735 0.957 0.000253 *** |
| OTU_166 0.956 0.001931 ** |
| OTU_722 0.956 0.000253 *** |
| OTU_1586 0.955 0.000253 *** |
| OTU_1141 0.955 0.000253 *** |
| OTU_1499 0.955 0.000253 *** |
| OTU_1329 0.954 0.000253 *** |
| OTU_340 0.953 0.000253 *** |
| OTU_334 0.953 0.000253 *** |
| OTU_1978 0.953 0.000253 *** |
| OTU_1583 0.950 0.000253 *** |
| OTU_299 0.950 0.000461 *** |
| OTU_902 0.950 0.000253 *** |
| OTU_1435 0.949 0.000253 *** |
| OTU_171 0.949 0.000253 *** |
| OTU_816 0.949 0.000253 *** |
| OTU_197 0.949 0.000253 *** |
| OTU_427 0.949 0.000253 *** |
| OTU_226 0.949 0.000253 *** |
| OTU_1778 0.949 0.000253 *** |
| OTU_669 0.949 0.000253 *** |
| OTU_188 0.949 0.000253 *** |
| OTU_2086 0.949 0.000253 *** |
| OTU_2911 0.949 0.000253 *** |
| OTU_2314 0.949 0.000253 *** |
| OTU_2396 0.949 0.000253 *** |
| OTU_4443 0.949 0.000253 *** |
| OTU_92 0.949 0.000253 *** |
| OTU_4169 0.949 0.000253 *** |
| OTU_795 0.949 0.000253 *** |
| OTU_8411 0.949 0.000253 *** |
| OTU_2529 0.949 0.000253 *** |
| OTU_1528 0.949 0.000253 *** |
| OTU_7600 0.949 0.000253 *** |
| OTU_346 0.949 0.000253 *** |
| OTU_2104 0.949 0.000253 *** |
| OTU_2244 0.949 0.000253 *** |
| OTU_2475 0.949 0.000253 *** |
| OTU_2787 0.949 0.000253 *** |
| OTU_1140 0.949 0.000253 *** |
| OTU_2109 0.949 0.000253 *** |
| OTU_11307 0.949 0.000253 *** |
| OTU_4723 0.949 0.000253 *** |
| OTU_3086 0.949 0.000253 *** |
| OTU_1561 0.949 0.000253 *** |
| OTU_479 0.949 0.000253 *** |
| OTU_2425 0.949 0.000253 *** |
| OTU_3407 0.949 0.000253 *** |
| OTU_9164 0.949 0.000253 *** |
| OTU_3365 0.949 0.000253 *** |
| OTU_4230 0.949 0.000253 *** |
| OTU_738 0.949 0.000253 *** |
| OTU_3835 0.949 0.000253 *** |
| OTU_569 0.949 0.000253 *** |
| OTU_1700 0.949 0.000253 *** |
| OTU_3959 0.949 0.000253 *** |
| OTU_2339 0.949 0.000253 *** |
| OTU_2179 0.949 0.000253 *** |
| OTU_3708 0.949 0.000253 *** |
| OTU_860 0.949 0.000253 *** |
| OTU_3029 0.949 0.000253 *** |
| OTU_420 0.949 0.000253 *** |
| OTU_2680 0.949 0.000253 *** |
| OTU_10532 0.949 0.000253 *** |
| OTU_2678 0.949 0.000253 *** |
| OTU_2398 0.949 0.000253 *** |
| OTU_712 0.949 0.000253 *** |
| OTU_600 0.949 0.000253 *** |
| OTU_5883 0.949 0.000253 *** |
| OTU_10210 0.949 0.000253 *** |
| OTU_2008 0.949 0.000253 *** |
| OTU_11635 0.949 0.000253 *** |
| OTU_3793 0.949 0.000253 *** |
| OTU_4134 0.949 0.000253 *** |
| OTU_11434 0.949 0.000253 *** |
| OTU_4448 0.949 0.000253 *** |
| OTU_2838 0.949 0.000253 *** |
| OTU_357 0.949 0.000253 *** |
| OTU_10844 0.949 0.000253 *** |
| OTU_2073 0.949 0.000253 *** |
| OTU_5457 0.949 0.000253 *** |
| OTU_2266 0.949 0.000253 *** |
| OTU_6024 0.949 0.000253 *** |
| OTU_10102 0.949 0.000253 *** |
| OTU_447 0.949 0.000253 *** |
| OTU_2362 0.949 0.000253 *** |
| OTU_2150 0.949 0.000253 *** |
| OTU_2934 0.949 0.000253 *** |
| OTU_7010 0.949 0.000253 *** |
| OTU_5418 0.949 0.000253 *** |
| OTU_6747 0.949 0.000253 *** |
| OTU_5535 0.949 0.000253 *** |
| OTU_6736 0.949 0.000253 *** |
| OTU_2588 0.949 0.000253 *** |
| OTU_272 0.949 0.000253 *** |
| OTU_3509 0.949 0.000253 *** |
| OTU_3166 0.949 0.000253 *** |
| OTU_2333 0.949 0.000253 *** |
| OTU_3761 0.949 0.000253 *** |
| OTU_2434 0.949 0.000253 *** |
| OTU_3108 0.949 0.000253 *** |
| OTU_4368 0.949 0.000253 *** |
| OTU_3634 0.949 0.000253 *** |
| OTU_1448 0.949 0.000253 *** |
| OTU_3130 0.949 0.000253 *** |
| OTU_2239 0.949 0.000253 *** |
| OTU_1279 0.949 0.000253 *** |
| OTU_4209 0.949 0.000253 *** |
| OTU_3622 0.949 0.000253 *** |
| OTU_5236 0.949 0.000253 *** |
| OTU_1188 0.949 0.000253 *** |
| OTU_1482 0.949 0.000253 *** |
| OTU_1967 0.949 0.000253 *** |
| OTU_2235 0.949 0.000253 *** |
| OTU_2384 0.949 0.000253 *** |
| OTU_2743 0.949 0.000253 *** |
| OTU_1656 0.949 0.000253 *** |
| OTU_2135 0.949 0.000253 *** |
| OTU_7244 0.949 0.000253 *** |
| OTU_3139 0.949 0.000253 *** |
| OTU_4734 0.949 0.000253 *** |
| OTU_875 0.949 0.000253 *** |
| OTU_6278 0.949 0.000253 *** |
| OTU_3504 0.949 0.000253 *** |
| OTU_2752 0.949 0.000253 *** |
| OTU_1177 0.949 0.000253 *** |
| OTU_3175 0.949 0.000253 *** |
| OTU_2579 0.949 0.000253 *** |
| OTU_2375 0.949 0.000253 *** |
| OTU_9782 0.949 0.000253 *** |
| OTU_9255 0.949 0.000253 *** |
| OTU_3821 0.949 0.000253 *** |
| OTU_1780 0.949 0.000253 *** |
| OTU_2719 0.949 0.000253 *** |
| OTU_2706 0.949 0.000253 *** |
| OTU_3683 0.949 0.000253 *** |
| OTU_3867 0.949 0.000253 *** |
| OTU_3762 0.949 0.000253 *** |
| OTU_4177 0.949 0.000253 *** |
| OTU_2075 0.949 0.000253 *** |
| OTU_5378 0.949 0.000253 *** |
| OTU_4471 0.949 0.000253 *** |
| OTU_2417 0.949 0.000253 *** |
| OTU_3513 0.949 0.000253 *** |
| OTU_3475 0.949 0.000253 *** |
| OTU_2392 0.949 0.000253 *** |
| OTU_6397 0.949 0.000253 *** |
| OTU_3559 0.949 0.000253 *** |
| OTU_4328 0.949 0.000253 *** |
| OTU_1653 0.949 0.000253 *** |
| OTU_3648 0.949 0.000253 *** |
| OTU_3074 0.949 0.000253 *** |
| OTU_4152 0.949 0.000253 *** |
| OTU_1541 0.949 0.000253 *** |
| OTU_2247 0.949 0.000253 *** |
| OTU_3093 0.949 0.000253 *** |
| OTU_2354 0.949 0.000253 *** |
| OTU_2947 0.949 0.000253 *** |
| OTU_3296 0.949 0.000253 *** |
| OTU_2770 0.949 0.000253 *** |
| OTU_3034 0.949 0.000253 *** |
| OTU_4630 0.949 0.000253 *** |
| OTU_2327 0.949 0.000253 *** |
| OTU_1535 0.949 0.000253 *** |
| OTU_2586 0.949 0.000253 *** |
| OTU_11387 0.949 0.000253 *** |
| OTU_1752 0.949 0.000253 *** |
| OTU_1581 0.949 0.000253 *** |
| OTU_3704 0.949 0.000253 *** |
| OTU_2822 0.949 0.000253 *** |
| OTU_2410 0.949 0.000253 *** |
| OTU_11227 0.949 0.000253 *** |
| OTU_2815 0.949 0.000253 *** |
| OTU_5169 0.949 0.000253 *** |
| OTU_3970 0.949 0.000253 *** |
| OTU_2522 0.949 0.000253 *** |
| OTU_4956 0.949 0.000253 *** |
| OTU_2140 0.949 0.000253 *** |
| OTU_1983 0.949 0.000253 *** |
| OTU_3698 0.949 0.000253 *** |
| OTU_3411 0.949 0.000253 *** |
| OTU_1500 0.949 0.000253 *** |
| OTU_3845 0.949 0.000253 *** |
| OTU_3227 0.949 0.000253 *** |
| OTU_2441 0.949 0.000253 *** |
| OTU_5560 0.949 0.000253 *** |
| OTU_4829 0.949 0.000253 *** |
| OTU_4930 0.949 0.000253 *** |
| OTU_1679 0.949 0.000253 *** |
| OTU_4752 0.949 0.000253 *** |
| OTU_2213 0.949 0.000253 *** |
| OTU_4942 0.949 0.000253 *** |
| OTU_8783 0.949 0.000253 *** |
| OTU_4121 0.949 0.000253 *** |
| OTU_12304 0.949 0.000253 *** |
| OTU_2726 0.949 0.000253 *** |
| OTU_4310 0.949 0.000253 *** |
| OTU_4713 0.949 0.000253 *** |
| OTU_4059 0.949 0.000253 *** |
| OTU_2817 0.949 0.000253 *** |
| OTU_4224 0.949 0.000253 *** |
| OTU_3766 0.949 0.000253 *** |
| OTU_2958 0.949 0.000253 *** |
| OTU_2163 0.949 0.000253 *** |
| OTU_3129 0.949 0.000253 *** |
| OTU_4589 0.949 0.000253 *** |
| OTU_3926 0.949 0.000253 *** |
| OTU_2747 0.949 0.000253 *** |
| OTU_3173 0.949 0.000253 *** |
| OTU_9561 0.949 0.000253 *** |
| OTU_4103 0.949 0.000253 *** |
| OTU_10303 0.949 0.000253 *** |
| OTU_2004 0.949 0.000253 *** |
| OTU_4259 0.949 0.000253 *** |
| OTU_2296 0.949 0.000253 *** |
| OTU_10761 0.949 0.000253 *** |
| OTU_4203 0.949 0.000253 *** |
| OTU_7417 0.949 0.000253 *** |
| OTU_5191 0.949 0.000253 *** |
| OTU_10540 0.949 0.000253 *** |
| OTU_5845 0.949 0.000253 *** |
| OTU_2819 0.949 0.000253 *** |
| OTU_6294 0.949 0.000253 *** |
| OTU_3220 0.949 0.000253 *** |
| OTU_3306 0.949 0.000253 *** |
| OTU_4300 0.949 0.000253 *** |
| OTU_4453 0.949 0.000253 *** |
| OTU_2210 0.949 0.000253 *** |
| OTU_3758 0.949 0.000253 *** |
| OTU_3257 0.949 0.000253 *** |
| OTU_5453 0.949 0.000253 *** |
| OTU_2667 0.949 0.000253 *** |
| OTU_4765 0.949 0.000253 *** |
| OTU_6571 0.949 0.000253 *** |
| OTU_8695 0.949 0.000253 *** |
| OTU_2778 0.949 0.000253 *** |
| OTU_3891 0.949 0.000253 *** |
| OTU_4696 0.949 0.000253 *** |
| OTU_3791 0.949 0.000253 *** |
| OTU_3600 0.949 0.000253 *** |
| OTU_2875 0.949 0.000253 *** |
| OTU_4112 0.949 0.000253 *** |
| OTU_3079 0.949 0.000253 *** |
| OTU_3215 0.949 0.000253 *** |
| OTU_3363 0.949 0.000253 *** |
| OTU_2629 0.949 0.000253 *** |
| OTU_2453 0.949 0.000253 *** |
| OTU_2055 0.949 0.000253 *** |
| OTU_11196 0.949 0.000253 *** |
| OTU_5402 0.949 0.000253 *** |
| OTU_2593 0.949 0.000253 *** |
| OTU_4406 0.949 0.000253 *** |
| OTU_4302 0.949 0.000253 *** |
| OTU_3962 0.949 0.000253 *** |
| OTU_8736 0.949 0.000253 *** |
| OTU_2533 0.949 0.000253 *** |
| OTU_4192 0.949 0.000253 *** |
| OTU_2899 0.949 0.000253 *** |
| OTU_3581 0.949 0.000253 *** |
| OTU_11477 0.949 0.000253 *** |
| OTU_4552 0.949 0.000253 *** |
| OTU_2741 0.949 0.000253 *** |
| OTU_2892 0.949 0.000253 *** |
| OTU_2123 0.949 0.000253 *** |
| OTU_3567 0.949 0.000253 *** |
| OTU_4080 0.949 0.000253 *** |
| OTU_2229 0.949 0.000253 *** |
| OTU_1940 0.949 0.000253 *** |
| OTU_3211 0.949 0.000253 *** |
| OTU_3463 0.949 0.000253 *** |
| OTU_5760 0.949 0.000253 *** |
| OTU_4959 0.949 0.000253 *** |
| OTU_2859 0.949 0.000253 *** |
| OTU_5568 0.949 0.000253 *** |
| OTU_3135 0.949 0.000253 *** |
| OTU_2157 0.949 0.000253 *** |
| OTU_4584 0.949 0.000253 *** |
| OTU_4172 0.949 0.000253 *** |
| OTU_3792 0.949 0.000253 *** |
| OTU_4222 0.949 0.000253 *** |
| OTU_4973 0.949 0.000253 *** |
| OTU_2717 0.949 0.000253 *** |
| OTU_6598 0.949 0.000253 *** |
| OTU_7127 0.949 0.000253 *** |
| OTU_5544 0.949 0.000253 *** |
| OTU_4710 0.949 0.000253 *** |
| OTU_6734 0.949 0.000253 *** |
| OTU_4199 0.949 0.000253 *** |
| OTU_5920 0.949 0.000253 *** |
| OTU_2855 0.949 0.000253 *** |
| OTU_3397 0.949 0.000253 *** |
| OTU_5001 0.949 0.000253 *** |
| OTU_4539 0.949 0.000253 *** |
| OTU_7561 0.949 0.000253 *** |
| OTU_10144 0.949 0.000253 *** |
| OTU_4216 0.949 0.000253 *** |
| OTU_3276 0.949 0.000253 *** |
| OTU_4291 0.949 0.000253 *** |
| OTU_2753 0.949 0.000253 *** |
| OTU_3667 0.949 0.000253 *** |
| OTU_4135 0.949 0.000253 *** |
| OTU_11973 0.949 0.000253 *** |
| OTU_3311 0.949 0.000253 *** |
| OTU_3864 0.949 0.000253 *** |
| OTU_4206 0.949 0.000253 *** |
| OTU_7964 0.949 0.000253 *** |
| OTU_4911 0.949 0.000253 *** |
| OTU_5307 0.949 0.000253 *** |
| OTU_9686 0.949 0.000253 *** |
| OTU_2842 0.949 0.000253 *** |
| OTU_3458 0.949 0.000253 *** |
| OTU_4085 0.949 0.000253 *** |
| OTU_3449 0.949 0.000253 *** |
| OTU_6016 0.949 0.000253 *** |
| OTU_3734 0.949 0.000253 *** |
| OTU_4133 0.949 0.000253 *** |
| OTU_3583 0.949 0.000253 *** |
| OTU_10181 0.949 0.000253 *** |
| OTU_3432 0.949 0.000253 *** |
| OTU_3371 0.949 0.000253 *** |
| OTU_10870 0.949 0.000253 *** |
| OTU_6560 0.949 0.000253 *** |
| OTU_4676 0.949 0.000253 *** |
| OTU_7023 0.949 0.000253 *** |
| OTU_4205 0.949 0.000253 *** |
| OTU_1539 0.948 0.000461 *** |
| OTU_4188 0.948 0.000253 *** |
| OTU_723 0.948 0.000253 *** |
| OTU_844 0.947 0.000253 *** |
| OTU_1060 0.947 0.000253 *** |
| OTU_7088 0.947 0.000253 *** |
| OTU_2059 0.947 0.000253 *** |
| OTU_1888 0.946 0.000253 *** |
| OTU_1756 0.946 0.000253 *** |
| OTU_1942 0.946 0.000253 *** |
| OTU_2283 0.946 0.000253 *** |
| OTU_1247 0.946 0.000253 *** |
| OTU_1334 0.946 0.000253 *** |
| OTU_1972 0.946 0.000253 *** |
| OTU_2509 0.946 0.000253 *** |
| OTU_2276 0.945 0.000253 *** |
| OTU_5230 0.945 0.000253 *** |
| OTU_1316 0.945 0.000253 *** |
| OTU_8127 0.945 0.000253 *** |
| OTU_1029 0.945 0.000253 *** |
| OTU_1847 0.945 0.000253 *** |
| OTU_3309 0.945 0.000253 *** |
| OTU_2872 0.945 0.000253 *** |
| OTU_980 0.945 0.000253 *** |
| OTU_1624 0.945 0.000253 *** |
| OTU_2225 0.945 0.000253 *** |
| OTU_3145 0.945 0.000253 *** |
| OTU_756 0.944 0.000461 *** |
| OTU_1475 0.944 0.000253 *** |
| OTU_1398 0.944 0.000253 *** |
| OTU_2554 0.944 0.000253 *** |
| OTU_2011 0.944 0.000253 *** |
| OTU_1524 0.944 0.000253 *** |
| OTU_4181 0.943 0.000253 *** |
| OTU_270 0.943 0.000253 *** |
| OTU_2483 0.943 0.000253 *** |
| OTU_2414 0.943 0.000253 *** |
| OTU_3315 0.943 0.000253 *** |
| OTU_3258 0.943 0.000253 *** |
| OTU_3091 0.943 0.000253 *** |
| OTU_2637 0.943 0.000253 *** |
| OTU_4296 0.943 0.000253 *** |
| OTU_2832 0.943 0.000253 *** |
| OTU_10367 0.943 0.000253 *** |
| OTU_2526 0.942 0.000253 *** |
| OTU_1297 0.942 0.000253 *** |
| OTU_512 0.942 0.000253 *** |
| OTU_4054 0.942 0.000253 *** |
| OTU_11758 0.942 0.000253 *** |
| OTU_3219 0.942 0.000253 *** |
| OTU_11223 0.942 0.000253 *** |
| OTU_2391 0.942 0.000253 *** |
| OTU_3982 0.942 0.000253 *** |
| OTU_583 0.942 0.000253 *** |
| OTU_1698 0.942 0.000253 *** |
| OTU_2541 0.941 0.000253 *** |
| OTU_2309 0.941 0.000253 *** |
| OTU_2861 0.941 0.000253 *** |
| OTU_10550 0.941 0.000253 *** |
| OTU_2308 0.941 0.000253 *** |
| OTU_4105 0.941 0.000253 *** |
| OTU_6793 0.941 0.000253 *** |
| OTU_1827 0.941 0.000253 *** |
| OTU_5765 0.940 0.000253 *** |
| OTU_1540 0.940 0.000461 *** |
| OTU_2148 0.940 0.000253 *** |
| OTU_1056 0.940 0.000253 *** |
| OTU_2848 0.940 0.000253 *** |
| OTU_2854 0.940 0.000253 *** |
| OTU_1598 0.940 0.000253 *** |
| OTU_10507 0.940 0.000253 *** |
| OTU_3775 0.940 0.000253 *** |
| OTU_3155 0.940 0.000253 *** |
| OTU_3052 0.940 0.000253 *** |
| OTU_9367 0.940 0.000253 *** |
| OTU_10405 0.940 0.000253 *** |
| OTU_9282 0.939 0.000253 *** |
| OTU_3892 0.939 0.000253 *** |
| OTU_3921 0.939 0.000253 *** |
| OTU_1552 0.939 0.000253 *** |
| OTU_2517 0.939 0.000253 *** |
| OTU_323 0.939 0.000253 *** |
| OTU_1022 0.938 0.000253 *** |
| OTU_967 0.938 0.000253 *** |
| OTU_7515 0.938 0.000253 *** |
| OTU_1899 0.938 0.000253 *** |
| OTU_4240 0.938 0.000253 *** |
| OTU_2730 0.938 0.000253 *** |
| OTU_3281 0.938 0.000253 *** |
| OTU_1565 0.937 0.000253 *** |
| OTU_2948 0.937 0.000253 *** |
| OTU_4378 0.937 0.000253 *** |
| OTU_632 0.937 0.000253 *** |
| OTU_2397 0.937 0.000253 *** |
| OTU_2291 0.937 0.000253 *** |
| OTU_5181 0.936 0.000253 *** |
| OTU_3966 0.936 0.000253 *** |
| OTU_3973 0.936 0.000253 *** |
| OTU_2097 0.936 0.000253 *** |
| OTU_952 0.936 0.000253 *** |
| OTU_1716 0.936 0.000253 *** |
| OTU_3327 0.936 0.000253 *** |
| OTU_1611 0.935 0.000253 *** |
| OTU_1863 0.935 0.000253 *** |
| OTU_2245 0.935 0.000253 *** |
| OTU_3595 0.935 0.000253 *** |
| OTU_3517 0.935 0.000253 *** |
| OTU_823 0.935 0.000461 *** |
| OTU_11911 0.935 0.000253 *** |
| OTU_1897 0.935 0.000253 *** |
| OTU_4095 0.934 0.000253 *** |
| OTU_3305 0.934 0.000253 *** |
| OTU_5224 0.934 0.000253 *** |
| OTU_1239 0.934 0.000253 *** |
| OTU_5428 0.934 0.000253 *** |
| OTU_4583 0.934 0.000253 *** |
| OTU_5043 0.934 0.000253 *** |
| OTU_5642 0.933 0.000461 *** |
| OTU_6781 0.933 0.000253 *** |
| OTU_1378 0.933 0.000253 *** |
| OTU_10298 0.932 0.000253 *** |
| OTU_5565 0.932 0.000253 *** |
| OTU_3859 0.932 0.000461 *** |
| OTU_1436 0.932 0.000253 *** |
| OTU_2619 0.932 0.000253 *** |
| OTU_2342 0.931 0.000253 *** |
| OTU_5529 0.931 0.000253 *** |
| OTU_1260 0.931 0.000253 *** |
| OTU_9281 0.931 0.000253 *** |
| OTU_3725 0.930 0.000253 *** |
| OTU_5826 0.930 0.000253 *** |
| OTU_8548 0.929 0.000253 *** |
| OTU_2423 0.929 0.000253 *** |
| OTU_2479 0.928 0.000253 *** |
| OTU_7064 0.928 0.000253 *** |
| OTU_2024 0.927 0.000253 *** |
| OTU_5726 0.927 0.000253 *** |
| OTU_294 0.927 0.000253 *** |
| OTU_955 0.926 0.000253 *** |
| OTU_3024 0.926 0.000253 *** |
| OTU_5171 0.926 0.000253 *** |
| OTU_3409 0.926 0.000253 *** |
| OTU_1305 0.926 0.000253 *** |
| OTU_10408 0.926 0.000253 *** |
| OTU_2313 0.926 0.000253 *** |
| OTU_127 0.925 0.000253 *** |
| OTU_939 0.925 0.000253 *** |
| OTU_9023 0.925 0.000461 *** |
| OTU_4932 0.925 0.000253 *** |
| OTU_2713 0.925 0.000253 *** |
| OTU_2759 0.924 0.000253 *** |
| OTU_1891 0.924 0.000253 *** |
| OTU_2943 0.924 0.000253 *** |
| OTU_3862 0.923 0.000253 *** |
| OTU_8914 0.923 0.000253 *** |
| OTU_8011 0.923 0.000253 *** |
| OTU_4474 0.923 0.000253 *** |
| OTU_4003 0.923 0.000253 *** |
| OTU_1973 0.921 0.000253 *** |
| OTU_2146 0.921 0.000253 *** |
| OTU_407 0.920 0.000253 *** |
| OTU_5316 0.919 0.000253 *** |
| OTU_2227 0.919 0.000253 *** |
| OTU_1551 0.918 0.000253 *** |
| OTU_2012 0.917 0.000461 *** |
| OTU_8172 0.917 0.000461 *** |
| OTU_1995 0.917 0.000253 *** |
| OTU_128 0.916 0.000461 *** |
| OTU_7007 0.916 0.001255 ** |
| OTU_3920 0.916 0.000253 *** |
| OTU_9006 0.913 0.000253 *** |
| OTU_3485 0.912 0.000253 *** |
| OTU_3812 0.911 0.000253 *** |
| OTU_6092 0.910 0.000847 *** |
| OTU_1381 0.910 0.000253 *** |
| OTU_2716 0.909 0.000253 *** |
| OTU_296 0.909 0.000847 *** |
| OTU_388 0.908 0.010513 * |
| OTU_9362 0.906 0.000253 *** |
| OTU_2282 0.904 0.000658 *** |
| OTU_227 0.902 0.001051 ** |
| OTU_3071 0.902 0.000253 *** |
| OTU_882 0.902 0.000253 *** |
| OTU_2849 0.900 0.000253 *** |
| OTU_2048 0.899 0.000461 *** |
| OTU_3492 0.899 0.000253 *** |
| OTU_7003 0.898 0.000461 *** |
| OTU_1203 0.898 0.002235 ** |
| OTU_1003 0.896 0.000461 *** |
| OTU_47 0.894 0.000253 *** |
| OTU_9857 0.894 0.000253 *** |
| OTU_4350 0.894 0.000253 *** |
| OTU_5520 0.894 0.000253 *** |
| OTU_411 0.894 0.000461 *** |
| OTU_138 0.894 0.000253 *** |
| OTU_1768 0.894 0.000253 *** |
| OTU_743 0.894 0.000461 *** |
| OTU_4456 0.894 0.000253 *** |
| OTU_1621 0.894 0.000253 *** |
| OTU_344 0.894 0.000253 *** |
| OTU_4544 0.894 0.000253 *** |
| OTU_3388 0.894 0.000253 *** |
| OTU_6011 0.894 0.000253 *** |
| OTU_5206 0.894 0.000253 *** |
| OTU_1410 0.894 0.000253 *** |
| OTU_3657 0.894 0.000253 *** |
| OTU_941 0.894 0.000253 *** |
| OTU_11045 0.894 0.000253 *** |
| OTU_4099 0.894 0.000253 *** |
| OTU_1323 0.894 0.000253 *** |
| OTU_12077 0.894 0.000253 *** |
| OTU_1617 0.894 0.000461 *** |
| OTU_1990 0.894 0.000253 *** |
| OTU_5954 0.894 0.000253 *** |
| OTU_409 0.894 0.000461 *** |
| OTU_6368 0.894 0.000253 *** |
| OTU_4118 0.894 0.000461 *** |
| OTU_1008 0.894 0.000253 *** |
| OTU_454 0.894 0.000253 *** |
| OTU_4198 0.894 0.000253 *** |
| OTU_7278 0.894 0.000253 *** |
| OTU_3250 0.894 0.000253 *** |
| OTU_1677 0.894 0.000461 *** |
| OTU_7034 0.894 0.000253 *** |
| OTU_4341 0.894 0.000253 *** |
| OTU_6034 0.894 0.000253 *** |
| OTU_2030 0.894 0.000253 *** |
| OTU_4739 0.894 0.000253 *** |
| OTU_2722 0.894 0.000461 *** |
| OTU_4861 0.894 0.000253 *** |
| OTU_4149 0.894 0.000253 *** |
| OTU_2531 0.894 0.000253 *** |
| OTU_8312 0.894 0.000253 *** |
| OTU_4405 0.894 0.000253 *** |
| OTU_3877 0.894 0.000253 *** |
| OTU_4381 0.894 0.000461 *** |
| OTU_6984 0.894 0.000253 *** |
| OTU_5950 0.894 0.000253 *** |
| OTU_2924 0.894 0.000461 *** |
| OTU_586 0.894 0.000253 *** |
| OTU_2275 0.894 0.000461 *** |
| OTU_4565 0.894 0.000253 *** |
| OTU_2723 0.894 0.000253 *** |
| OTU_2169 0.894 0.000253 *** |
| OTU_4553 0.894 0.000461 *** |
| OTU_8615 0.894 0.000461 *** |
| OTU_3124 0.894 0.000253 *** |
| OTU_4027 0.894 0.000253 *** |
| OTU_3497 0.894 0.000253 *** |
| OTU_3751 0.894 0.000253 *** |
| OTU_2962 0.894 0.000253 *** |
| OTU_4093 0.894 0.000461 *** |
| OTU_4481 0.894 0.000253 *** |
| OTU_2731 0.894 0.000253 *** |
| OTU_2710 0.894 0.000461 *** |
| OTU_6060 0.894 0.000253 *** |
| OTU_1907 0.894 0.000253 *** |
| OTU_1770 0.894 0.000461 *** |
| OTU_4256 0.894 0.000253 *** |
| OTU_2411 0.894 0.000253 *** |
| OTU_2628 0.894 0.000461 *** |
| OTU_3068 0.894 0.000461 *** |
| OTU_2769 0.894 0.000253 *** |
| OTU_10422 0.894 0.000253 *** |
| OTU_2913 0.894 0.000253 *** |
| OTU_7762 0.894 0.000253 *** |
| OTU_3085 0.894 0.000253 *** |
| OTU_1585 0.894 0.000253 *** |
| OTU_3679 0.894 0.000253 *** |
| OTU_3563 0.894 0.000253 *** |
| OTU_3863 0.894 0.000461 *** |
| OTU_3702 0.894 0.000253 *** |
| OTU_2590 0.894 0.000253 *** |
| OTU_3800 0.894 0.000253 *** |
| OTU_3701 0.894 0.000253 *** |
| OTU_2591 0.894 0.000461 *** |
| OTU_4345 0.894 0.000253 *** |
| OTU_3677 0.894 0.000461 *** |
| OTU_10119 0.894 0.000461 *** |
| OTU_2457 0.894 0.000253 *** |
| OTU_3357 0.894 0.000253 *** |
| OTU_3854 0.894 0.000253 *** |
| OTU_5019 0.894 0.000253 *** |
| OTU_7101 0.894 0.000253 *** |
| OTU_3190 0.894 0.000253 *** |
| OTU_1542 0.894 0.000253 *** |
| OTU_1751 0.894 0.000253 *** |
| OTU_3784 0.894 0.000253 *** |
| OTU_2188 0.894 0.000253 *** |
| OTU_4270 0.894 0.000253 *** |
| OTU_4670 0.894 0.000461 *** |
| OTU_3345 0.894 0.000461 *** |
| OTU_2323 0.894 0.000253 *** |
| OTU_12275 0.894 0.000253 *** |
| OTU_6158 0.894 0.000461 *** |
| OTU_2472 0.894 0.000253 *** |
| OTU_1061 0.894 0.000253 *** |
| OTU_2259 0.894 0.000253 *** |
| OTU_8534 0.894 0.000253 *** |
| OTU_4110 0.894 0.000253 *** |
| OTU_10221 0.894 0.000461 *** |
| OTU_4950 0.894 0.000253 *** |
| OTU_3968 0.894 0.000461 *** |
| OTU_1852 0.894 0.000461 *** |
| OTU_2652 0.894 0.000461 *** |
| OTU_1623 0.894 0.000253 *** |
| OTU_3244 0.894 0.000253 *** |
| OTU_4331 0.894 0.000253 *** |
| OTU_4971 0.894 0.000253 *** |
| OTU_5078 0.894 0.000461 *** |
| OTU_2954 0.894 0.000461 *** |
| OTU_4062 0.894 0.000253 *** |
| OTU_4543 0.894 0.000253 *** |
| OTU_2811 0.894 0.000253 *** |
| OTU_9408 0.894 0.000253 *** |
| OTU_9365 0.894 0.000461 *** |
| OTU_2797 0.894 0.000253 *** |
| OTU_5423 0.894 0.000253 *** |
| OTU_5949 0.894 0.000253 *** |
| OTU_12233 0.894 0.000253 *** |
| OTU_6106 0.894 0.000253 *** |
| OTU_4717 0.894 0.000253 *** |
| OTU_7892 0.894 0.000461 *** |
| OTU_2028 0.894 0.000253 *** |
| OTU_2676 0.894 0.000253 *** |
| OTU_2862 0.894 0.000253 *** |
| OTU_2060 0.894 0.000253 *** |
| OTU_7811 0.894 0.000253 *** |
| OTU_5117 0.894 0.000253 *** |
| OTU_2865 0.894 0.000253 *** |
| OTU_6337 0.894 0.000253 *** |
| OTU_3579 0.894 0.000253 *** |
| OTU_2420 0.894 0.000461 *** |
| OTU_8212 0.894 0.000253 *** |
| OTU_4693 0.894 0.000253 *** |
| OTU_2082 0.894 0.000253 *** |
| OTU_5315 0.894 0.000253 *** |
| OTU_9865 0.894 0.000253 *** |
| OTU_4656 0.894 0.000253 *** |
| OTU_3131 0.894 0.000253 *** |
| OTU_3870 0.894 0.000253 *** |
| OTU_3783 0.894 0.000253 *** |
| OTU_4241 0.894 0.000253 *** |
| OTU_7706 0.894 0.000253 *** |
| OTU_2508 0.894 0.000253 *** |
| OTU_2460 0.894 0.000253 *** |
| OTU_6725 0.894 0.000253 *** |
| OTU_4414 0.894 0.000253 *** |
| OTU_4914 0.894 0.000253 *** |
| OTU_6870 0.894 0.000253 *** |
| OTU_3873 0.894 0.000253 *** |
| OTU_5212 0.894 0.000253 *** |
| OTU_7171 0.894 0.000253 *** |
| OTU_10169 0.894 0.000461 *** |
| OTU_4092 0.894 0.000253 *** |
| OTU_11331 0.894 0.000253 *** |
| OTU_2839 0.894 0.000253 *** |
| OTU_2131 0.894 0.000253 *** |
| OTU_5180 0.894 0.000461 *** |
| OTU_2984 0.894 0.000253 *** |
| OTU_9352 0.894 0.000253 *** |
| OTU_5114 0.894 0.000253 *** |
| OTU_2978 0.894 0.000461 *** |
| OTU_4809 0.894 0.000253 *** |
| OTU_5177 0.894 0.000253 *** |
| OTU_4266 0.894 0.000253 *** |
| OTU_3961 0.894 0.000253 *** |
| OTU_3743 0.894 0.000461 *** |
| OTU_1989 0.894 0.000253 *** |
| OTU_4536 0.894 0.000253 *** |
| OTU_10880 0.894 0.000253 *** |
| OTU_6082 0.894 0.000253 *** |
| OTU_2538 0.894 0.000461 *** |
| OTU_2647 0.894 0.000253 *** |
| OTU_9646 0.894 0.000461 *** |
| OTU_10816 0.894 0.000253 *** |
| OTU_8171 0.894 0.000461 *** |
| OTU_7583 0.894 0.000461 *** |
| OTU_6032 0.894 0.000461 *** |
| OTU_11278 0.894 0.000461 *** |
| OTU_4522 0.894 0.000253 *** |
| OTU_3290 0.894 0.000253 *** |
| OTU_3324 0.894 0.000253 *** |
| OTU_4012 0.894 0.000253 *** |
| OTU_9385 0.894 0.000253 *** |
| OTU_9128 0.894 0.000253 *** |
| OTU_5415 0.894 0.000253 *** |
| OTU_12098 0.894 0.000253 *** |
| OTU_3430 0.894 0.000461 *** |
| OTU_3297 0.894 0.000461 *** |
| OTU_4473 0.894 0.000253 *** |
| OTU_3871 0.894 0.000253 *** |
| OTU_6355 0.894 0.000253 *** |
| OTU_4388 0.894 0.000253 *** |
| OTU_9347 0.894 0.000253 *** |
| OTU_7404 0.894 0.000253 *** |
| OTU_2765 0.894 0.000253 *** |
| OTU_5571 0.894 0.000253 *** |
| OTU_5454 0.894 0.000253 *** |
| OTU_6584 0.894 0.000253 *** |
| OTU_4976 0.894 0.000253 *** |
| OTU_3089 0.894 0.000253 *** |
| OTU_7556 0.894 0.000253 *** |
| OTU_5812 0.894 0.000461 *** |
| OTU_4619 0.894 0.000253 *** |
| OTU_8905 0.894 0.000253 *** |
| OTU_6028 0.894 0.000253 *** |
| OTU_4689 0.894 0.000253 *** |
| OTU_2870 0.894 0.000253 *** |
| OTU_3412 0.894 0.000461 *** |
| OTU_4960 0.894 0.000253 *** |
| OTU_10875 0.894 0.000461 *** |
| OTU_2885 0.894 0.000253 *** |
| OTU_4063 0.894 0.000253 *** |
| OTU_5107 0.894 0.000253 *** |
| OTU_8645 0.894 0.000253 *** |
| OTU_4519 0.894 0.000253 *** |
| OTU_4763 0.894 0.000253 *** |
| OTU_4870 0.894 0.000461 *** |
| OTU_2373 0.894 0.000253 *** |
| OTU_7299 0.894 0.000253 *** |
| OTU_3415 0.894 0.000253 *** |
| OTU_8228 0.894 0.000253 *** |
| OTU_3846 0.894 0.000253 *** |
| OTU_4219 0.894 0.000461 *** |
| OTU_6802 0.894 0.000253 *** |
| OTU_4215 0.894 0.000461 *** |
| OTU_7295 0.894 0.000253 *** |
| OTU_4267 0.894 0.000253 *** |
| OTU_5554 0.894 0.000253 *** |
| OTU_4745 0.894 0.000253 *** |
| OTU_3237 0.894 0.000461 *** |
| OTU_9363 0.894 0.000253 *** |
| OTU_4097 0.894 0.000461 *** |
| OTU_5226 0.894 0.000461 *** |
| OTU_5861 0.894 0.000253 *** |
| OTU_3785 0.894 0.000253 *** |
| OTU_6247 0.894 0.000253 *** |
| OTU_10254 0.894 0.000253 *** |
| OTU_4887 0.894 0.000253 *** |
| OTU_8405 0.894 0.000253 *** |
| OTU_4672 0.894 0.000253 *** |
| OTU_3568 0.894 0.000253 *** |
| OTU_4546 0.894 0.000253 *** |
| OTU_4277 0.894 0.000253 *** |
| OTU_3342 0.894 0.000253 *** |
| OTU_3884 0.894 0.000253 *** |
| OTU_5326 0.894 0.000253 *** |
| OTU_4282 0.894 0.000253 *** |
| OTU_7077 0.894 0.000461 *** |
| OTU_5882 0.894 0.000461 *** |
| OTU_4665 0.894 0.000461 *** |
| OTU_5027 0.894 0.000253 *** |
| OTU_4962 0.894 0.000253 *** |
| OTU_4825 0.894 0.000253 *** |
| OTU_6269 0.894 0.000461 *** |
| OTU_4903 0.894 0.000253 *** |
| OTU_4064 0.894 0.000461 *** |
| OTU_4362 0.894 0.000253 *** |
| OTU_7234 0.894 0.000253 *** |
| OTU_3838 0.894 0.000253 *** |
| OTU_10022 0.894 0.000253 *** |
| OTU_7070 0.894 0.000253 *** |
| OTU_598 0.894 0.000253 *** |
| OTU_34 0.893 0.000658 *** |
| OTU_564 0.892 0.000461 *** |
| OTU_2557 0.891 0.000253 *** |
| OTU_1345 0.890 0.000253 *** |
| OTU_1427 0.890 0.000461 *** |
| OTU_3136 0.890 0.000253 *** |
| OTU_2170 0.890 0.000253 *** |
| OTU_2395 0.890 0.000253 *** |
| OTU_2149 0.890 0.000253 *** |
| OTU_3451 0.890 0.000253 *** |
| OTU_2955 0.890 0.000253 *** |
| OTU_2740 0.890 0.000253 *** |
| OTU_1894 0.889 0.000461 *** |
| OTU_2687 0.889 0.000253 *** |
| OTU_1517 0.889 0.000253 *** |
| OTU_11578 0.889 0.000461 *** |
| OTU_2729 0.889 0.000461 *** |
| OTU_2860 0.888 0.000253 *** |
| OTU_2863 0.888 0.000461 *** |
| OTU_3620 0.888 0.000253 *** |
| OTU_616 0.888 0.000253 *** |
| OTU_1873 0.888 0.000253 *** |
| OTU_5002 0.888 0.000253 *** |
| OTU_3639 0.888 0.000253 *** |
| OTU_4523 0.888 0.000253 *** |
| OTU_3988 0.888 0.000253 *** |
| OTU_1223 0.888 0.000658 *** |
| OTU_7368 0.887 0.000253 *** |
| OTU_3113 0.887 0.002894 ** |
| OTU_3159 0.887 0.000658 *** |
| OTU_2945 0.887 0.000461 *** |
| OTU_2627 0.887 0.000461 *** |
| OTU_5115 0.886 0.000253 *** |
| OTU_3090 0.886 0.000253 *** |
| OTU_4766 0.886 0.000461 *** |
| OTU_4867 0.886 0.000461 *** |
| OTU_4038 0.886 0.000253 *** |
| OTU_2197 0.886 0.000253 *** |
| OTU_1105 0.886 0.005272 ** |
| OTU_2938 0.886 0.000253 *** |
| OTU_708 0.885 0.000461 *** |
| OTU_12128 0.885 0.000461 *** |
| OTU_2896 0.885 0.000253 *** |
| OTU_4509 0.885 0.000253 *** |
| OTU_4011 0.884 0.000253 *** |
| OTU_4402 0.884 0.000658 *** |
| OTU_7935 0.884 0.000253 *** |
| OTU_2622 0.883 0.000253 *** |
| OTU_4615 0.883 0.000461 *** |
| OTU_3678 0.883 0.000461 *** |
| OTU_3755 0.882 0.000461 *** |
| OTU_3945 0.882 0.000461 *** |
| OTU_4633 0.882 0.000658 *** |
| OTU_3869 0.881 0.000461 *** |
| OTU_2993 0.881 0.000658 *** |
| OTU_10009 0.881 0.000253 *** |
| OTU_3487 0.881 0.000847 *** |
| OTU_3827 0.881 0.000461 *** |
| OTU_4592 0.880 0.000658 *** |
| OTU_5709 0.880 0.000253 *** |
| OTU_3659 0.880 0.000461 *** |
| OTU_2390 0.880 0.000461 *** |
| OTU_4096 0.880 0.000253 *** |
| OTU_7499 0.880 0.000461 *** |
| OTU_2959 0.880 0.000658 *** |
| OTU_4855 0.879 0.000461 *** |
| OTU_3760 0.879 0.000253 *** |
| OTU_3012 0.878 0.000461 *** |
| OTU_2106 0.877 0.000461 *** |
| OTU_4285 0.877 0.000658 *** |
| OTU_4265 0.876 0.000253 *** |
| OTU_3066 0.876 0.000658 *** |
| OTU_5606 0.875 0.000658 *** |
| OTU_6184 0.875 0.000658 *** |
| OTU_4537 0.875 0.000461 *** |
| OTU_2119 0.875 0.000847 *** |
| OTU_5017 0.874 0.000253 *** |
| OTU_9172 0.874 0.000461 *** |
| OTU_1557 0.873 0.000461 *** |
| OTU_8413 0.873 0.000253 *** |
| OTU_6877 0.873 0.000658 *** |
| OTU_6519 0.873 0.000253 *** |
| OTU_4679 0.873 0.000461 *** |
| OTU_4392 0.873 0.000461 *** |
| OTU_339 0.871 0.000658 *** |
| OTU_163 0.871 0.001255 ** |
| OTU_6041 0.871 0.000847 *** |
| OTU_1671 0.870 0.000658 *** |
| OTU_2501 0.870 0.000658 *** |
| OTU_9757 0.870 0.000658 *** |
| OTU_9065 0.870 0.000253 *** |
| OTU_8497 0.867 0.000461 *** |
| OTU_5008 0.866 0.000461 *** |
| OTU_2463 0.865 0.000253 *** |
| OTU_2818 0.865 0.000847 *** |
| OTU_5746 0.864 0.000253 *** |
| OTU_822 0.864 0.000461 *** |
| OTU_369 0.864 0.000253 *** |
| OTU_7724 0.864 0.000461 *** |
| OTU_5390 0.863 0.000658 *** |
| OTU_1900 0.862 0.000461 *** |
| OTU_9843 0.862 0.000461 *** |
| OTU_244 0.862 0.001594 ** |
| OTU_11687 0.862 0.000658 *** |
| OTU_3731 0.861 0.000658 *** |
| OTU_3893 0.861 0.000253 *** |
| OTU_4201 0.860 0.000461 *** |
| OTU_1207 0.860 0.000658 *** |
| OTU_2316 0.855 0.000461 *** |
| OTU_1026 0.854 0.001440 ** |
| OTU_7185 0.851 0.000253 *** |
| OTU_724 0.849 0.009235 ** |
| OTU_1949 0.844 0.001594 ** |
| OTU_2464 0.842 0.001440 ** |
| OTU_778 0.841 0.000253 *** |
| OTU_2102 0.840 0.000658 *** |
| OTU_863 0.840 0.007600 ** |
| OTU_3179 0.839 0.001051 ** |
| OTU_3413 0.838 0.000658 *** |
| OTU_3291 0.837 0.000658 *** |
| OTU_1994 0.837 0.000253 *** |
| OTU_574 0.837 0.000658 *** |
| OTU_702 0.837 0.000461 *** |
| OTU_12242 0.837 0.000253 *** |
| OTU_10234 0.837 0.000253 *** |
| OTU_273 0.837 0.000253 *** |
| OTU_978 0.837 0.000847 *** |
| OTU_5439 0.837 0.000847 *** |
| OTU_8388 0.837 0.000847 *** |
| OTU_5055 0.837 0.000847 *** |
| OTU_596 0.837 0.000253 *** |
| OTU_1521 0.837 0.000658 *** |
| OTU_3500 0.837 0.000658 *** |
| OTU_4699 0.837 0.000253 *** |
| OTU_4836 0.837 0.000253 *** |
| OTU_1757 0.837 0.000461 *** |
| OTU_2798 0.837 0.000461 *** |
| OTU_2495 0.837 0.000658 *** |
| OTU_4089 0.837 0.000253 *** |
| OTU_1445 0.837 0.000847 *** |
| OTU_467 0.837 0.000461 *** |
| OTU_9239 0.837 0.000253 *** |
| OTU_2158 0.837 0.000847 *** |
| OTU_2196 0.837 0.000253 *** |
| OTU_5182 0.837 0.000461 *** |
| OTU_5044 0.837 0.000253 *** |
| OTU_766 0.837 0.000658 *** |
| OTU_4812 0.837 0.001255 ** |
| OTU_10332 0.837 0.000461 *** |
| OTU_4641 0.837 0.001051 ** |
| OTU_4767 0.837 0.000461 *** |
| OTU_3967 0.837 0.000253 *** |
| OTU_551 0.837 0.000461 *** |
| OTU_5468 0.837 0.000461 *** |
| OTU_5090 0.837 0.000461 *** |
| OTU_4542 0.837 0.000253 *** |
| OTU_1688 0.837 0.000847 *** |
| OTU_1234 0.837 0.000658 *** |
| OTU_5895 0.837 0.000253 *** |
| OTU_4210 0.837 0.000847 *** |
| OTU_3868 0.837 0.001051 ** |
| OTU_6784 0.837 0.000847 *** |
| OTU_1418 0.837 0.000847 *** |
| OTU_3954 0.837 0.000658 *** |
| OTU_5749 0.837 0.000253 *** |
| OTU_2548 0.837 0.000461 *** |
| OTU_5420 0.837 0.000658 *** |
| OTU_10202 0.837 0.000253 *** |
| OTU_11444 0.837 0.000847 *** |
| OTU_2774 0.837 0.000253 *** |
| OTU_2654 0.837 0.000461 *** |
| OTU_5706 0.837 0.000461 *** |
| OTU_3547 0.837 0.000461 *** |
| OTU_8367 0.837 0.000461 *** |
| OTU_3724 0.837 0.000847 *** |
| OTU_2101 0.837 0.000658 *** |
| OTU_2980 0.837 0.000253 *** |
| OTU_4857 0.837 0.000658 *** |
| OTU_588 0.837 0.000461 *** |
| OTU_1607 0.837 0.000253 *** |
| OTU_3850 0.837 0.000658 *** |
| OTU_3193 0.837 0.000658 *** |
| OTU_4306 0.837 0.000461 *** |
| OTU_5985 0.837 0.000658 *** |
| OTU_5189 0.837 0.000658 *** |
| OTU_4883 0.837 0.000658 *** |
| OTU_1616 0.837 0.000658 *** |
| OTU_2232 0.837 0.000658 *** |
| OTU_2519 0.837 0.000253 *** |
| OTU_5102 0.837 0.000658 *** |
| OTU_10142 0.837 0.000658 *** |
| OTU_3361 0.837 0.000253 *** |
| OTU_2357 0.837 0.000658 *** |
| OTU_4663 0.837 0.000461 *** |
| OTU_1820 0.837 0.000461 *** |
| OTU_8235 0.837 0.000461 *** |
| OTU_8918 0.837 0.001051 ** |
| OTU_1610 0.837 0.000658 *** |
| OTU_3301 0.837 0.000253 *** |
| OTU_2971 0.837 0.000253 *** |
| OTU_4482 0.837 0.000658 *** |
| OTU_2280 0.837 0.000658 *** |
| OTU_4354 0.837 0.000253 *** |
| OTU_11029 0.837 0.000253 *** |
| OTU_7329 0.837 0.001051 ** |
| OTU_5407 0.837 0.000847 *** |
| OTU_2966 0.837 0.000461 *** |
| OTU_2999 0.837 0.000658 *** |
| OTU_3814 0.837 0.000847 *** |
| OTU_6522 0.837 0.000847 *** |
| OTU_6048 0.837 0.000253 *** |
| OTU_3865 0.837 0.000461 *** |
| OTU_3084 0.837 0.000461 *** |
| OTU_3514 0.837 0.000253 *** |
| OTU_3230 0.837 0.000253 *** |
| OTU_5087 0.837 0.000847 *** |
| OTU_2406 0.837 0.000253 *** |
| OTU_5427 0.837 0.000461 *** |
| OTU_6174 0.837 0.000461 *** |
| OTU_3030 0.837 0.000658 *** |
| OTU_5066 0.837 0.000253 *** |
| OTU_6132 0.837 0.000658 *** |
| OTU_4832 0.837 0.000847 *** |
| OTU_4585 0.837 0.000847 *** |
| OTU_3312 0.837 0.000253 *** |
| OTU_4072 0.837 0.000847 *** |
| OTU_1154 0.837 0.000461 *** |
| OTU_6643 0.837 0.000253 *** |
| OTU_4491 0.837 0.000461 *** |
| OTU_3598 0.837 0.000658 *** |
| OTU_7008 0.837 0.000847 *** |
| OTU_6709 0.837 0.000461 *** |
| OTU_2721 0.837 0.000461 *** |
| OTU_4966 0.837 0.000461 *** |
| OTU_4963 0.837 0.000253 *** |
| OTU_3261 0.837 0.000253 *** |
| OTU_3593 0.837 0.000253 *** |
| OTU_3684 0.837 0.000658 *** |
| OTU_6156 0.837 0.000253 *** |
| OTU_2957 0.837 0.000253 *** |
| OTU_4278 0.837 0.000461 *** |
| OTU_5460 0.837 0.000253 *** |
| OTU_3378 0.837 0.000253 *** |
| OTU_6939 0.837 0.000253 *** |
| OTU_5220 0.837 0.000658 *** |
| OTU_3853 0.837 0.000253 *** |
| OTU_1508 0.837 0.000253 *** |
| OTU_2582 0.837 0.000461 *** |
| OTU_6809 0.837 0.000253 *** |
| OTU_2940 0.837 0.001255 ** |
| OTU_4570 0.837 0.000658 *** |
| OTU_6640 0.837 0.000461 *** |
| OTU_5450 0.837 0.000658 *** |
| OTU_3465 0.837 0.000461 *** |
| OTU_5502 0.837 0.000847 *** |
| OTU_3655 0.837 0.000658 *** |
| OTU_3406 0.837 0.000847 *** |
| OTU_3072 0.837 0.000253 *** |
| OTU_4316 0.837 0.000253 *** |
| OTU_9001 0.837 0.000253 *** |
| OTU_5759 0.837 0.000461 *** |
| OTU_5856 0.837 0.000658 *** |
| OTU_5399 0.837 0.000461 *** |
| OTU_4980 0.837 0.000658 *** |
| OTU_8283 0.837 0.000253 *** |
| OTU_5790 0.837 0.000658 *** |
| OTU_4028 0.837 0.000658 *** |
| OTU_10025 0.837 0.000461 *** |
| OTU_5164 0.837 0.000847 *** |
| OTU_5941 0.837 0.000253 *** |
| OTU_8857 0.837 0.000847 *** |
| OTU_2964 0.837 0.000253 *** |
| OTU_4229 0.837 0.000847 *** |
| OTU_10685 0.837 0.001051 ** |
| OTU_5509 0.837 0.000847 *** |
| OTU_3638 0.837 0.000847 *** |
| OTU_3705 0.837 0.000461 *** |
| OTU_4244 0.837 0.000253 *** |
| OTU_6398 0.837 0.000658 *** |
| OTU_2440 0.837 0.000253 *** |
| OTU_3289 0.837 0.000847 *** |
| OTU_4218 0.837 0.000461 *** |
| OTU_3454 0.837 0.000658 *** |
| OTU_5431 0.837 0.000658 *** |
| OTU_9970 0.837 0.000658 *** |
| OTU_7447 0.837 0.000253 *** |
| OTU_7542 0.837 0.000658 *** |
| OTU_6193 0.837 0.000461 *** |
| OTU_4228 0.837 0.000253 *** |
| OTU_6754 0.837 0.000658 *** |
| OTU_3904 0.837 0.000253 *** |
| OTU_2804 0.837 0.000461 *** |
| OTU_9894 0.837 0.000847 *** |
| OTU_3478 0.837 0.000253 *** |
| OTU_5647 0.837 0.000847 *** |
| OTU_5543 0.837 0.000847 *** |
| OTU_7533 0.837 0.000461 *** |
| OTU_4597 0.837 0.000847 *** |
| OTU_5886 0.837 0.000847 *** |
| OTU_12152 0.837 0.000658 *** |
| OTU_7842 0.837 0.000253 *** |
| OTU_1929 0.837 0.000461 *** |
| OTU_4435 0.837 0.000253 *** |
| OTU_9055 0.837 0.000658 *** |
| OTU_3120 0.837 0.000658 *** |
| OTU_7334 0.837 0.000253 *** |
| OTU_6492 0.837 0.000461 *** |
| OTU_5306 0.837 0.000461 *** |
| OTU_2949 0.837 0.000847 *** |
| OTU_4374 0.837 0.000658 *** |
| OTU_5023 0.837 0.000461 *** |
| OTU_7261 0.837 0.000658 *** |
| OTU_5217 0.837 0.000253 *** |
| OTU_3555 0.837 0.000461 *** |
| OTU_5250 0.837 0.000461 *** |
| OTU_6437 0.837 0.000847 *** |
| OTU_3875 0.837 0.000847 *** |
| OTU_6384 0.837 0.000658 *** |
| OTU_8609 0.837 0.000847 *** |
| OTU_4923 0.837 0.000847 *** |
| OTU_3402 0.837 0.000253 *** |
| OTU_7153 0.837 0.000658 *** |
| OTU_7738 0.837 0.000253 *** |
| OTU_4924 0.837 0.000847 *** |
| OTU_4806 0.837 0.000847 *** |
| OTU_5074 0.837 0.000658 *** |
| OTU_3631 0.837 0.000847 *** |
| OTU_9060 0.837 0.000658 *** |
| OTU_4101 0.837 0.000253 *** |
| OTU_6108 0.837 0.000847 *** |
| OTU_4347 0.837 0.000847 *** |
| OTU_3418 0.837 0.000253 *** |
| OTU_4810 0.837 0.000253 *** |
| OTU_6135 0.837 0.000847 *** |
| OTU_12058 0.837 0.000253 *** |
| OTU_4424 0.837 0.000658 *** |
| OTU_4194 0.837 0.000658 *** |
| OTU_4876 0.837 0.000253 *** |
| OTU_4170 0.837 0.000658 *** |
| OTU_8161 0.837 0.000253 *** |
| OTU_7085 0.837 0.000461 *** |
| OTU_7497 0.837 0.000461 *** |
| OTU_5433 0.837 0.000847 *** |
| OTU_8994 0.837 0.000847 *** |
| OTU_6451 0.837 0.000461 *** |
| OTU_3577 0.837 0.000658 *** |
| OTU_5663 0.837 0.000847 *** |
| OTU_5218 0.837 0.000461 *** |
| OTU_3976 0.837 0.000847 *** |
| OTU_3881 0.837 0.000847 *** |
| OTU_3385 0.837 0.000847 *** |
| OTU_6454 0.837 0.000461 *** |
| OTU_5556 0.837 0.000847 *** |
| OTU_6344 0.837 0.000461 *** |
| OTU_7362 0.837 0.000253 *** |
| OTU_7936 0.837 0.000658 *** |
| OTU_3571 0.837 0.000658 *** |
| OTU_5538 0.837 0.000658 *** |
| OTU_5349 0.837 0.000461 *** |
| OTU_8771 0.837 0.000658 *** |
| OTU_5084 0.837 0.000253 *** |
| OTU_5944 0.837 0.000461 *** |
| OTU_6340 0.837 0.000253 *** |
| OTU_4488 0.837 0.000461 *** |
| OTU_4497 0.837 0.000847 *** |
| OTU_8637 0.837 0.000847 *** |
| OTU_6002 0.837 0.000461 *** |
| OTU_3597 0.837 0.000847 *** |
| OTU_3002 0.837 0.000461 *** |
| OTU_4031 0.837 0.000658 *** |
| OTU_6663 0.837 0.000658 *** |
| OTU_5176 0.837 0.000847 *** |
| OTU_5503 0.837 0.000461 *** |
| OTU_3707 0.837 0.000461 *** |
| OTU_4144 0.837 0.000658 *** |
| OTU_3745 0.837 0.000253 *** |
| OTU_5368 0.837 0.000658 *** |
| OTU_3558 0.837 0.000461 *** |
| OTU_7686 0.837 0.000847 *** |
| OTU_3240 0.837 0.000461 *** |
| OTU_4171 0.837 0.000847 *** |
| OTU_6881 0.837 0.000847 *** |
| OTU_6374 0.837 0.000461 *** |
| OTU_4398 0.837 0.000253 *** |
| OTU_3944 0.837 0.000847 *** |
| OTU_3201 0.837 0.000658 *** |
| OTU_4286 0.837 0.000658 *** |
| OTU_7618 0.837 0.000461 *** |
| OTU_6996 0.837 0.000253 *** |
| OTU_5233 0.837 0.000847 *** |
| OTU_7988 0.837 0.000658 *** |
| OTU_5451 0.837 0.000847 *** |
| OTU_9173 0.837 0.000658 *** |
| OTU_5522 0.837 0.000847 *** |
| OTU_5558 0.837 0.000253 *** |
| OTU_3716 0.837 0.000658 *** |
| OTU_5391 0.837 0.000658 *** |
| OTU_3404 0.837 0.000658 *** |
| OTU_6230 0.837 0.000253 *** |
| OTU_5683 0.837 0.000658 *** |
| OTU_9673 0.837 0.000461 *** |
| OTU_4732 0.837 0.000461 *** |
| OTU_3619 0.837 0.001051 ** |
| OTU_4220 0.837 0.001051 ** |
| OTU_4386 0.837 0.000658 *** |
| OTU_6062 0.837 0.000658 *** |
| OTU_5091 0.837 0.000253 *** |
| OTU_11301 0.837 0.000461 *** |
| OTU_5267 0.837 0.000461 *** |
| OTU_6748 0.837 0.000847 *** |
| OTU_7612 0.837 0.000253 *** |
| OTU_6706 0.837 0.000253 *** |
| OTU_4023 0.837 0.000253 *** |
| OTU_7672 0.837 0.000658 *** |
| OTU_4255 0.837 0.001051 ** |
| OTU_3266 0.837 0.000847 *** |
| OTU_8390 0.837 0.000461 *** |
| OTU_6338 0.837 0.000847 *** |
| OTU_4048 0.837 0.000253 *** |
| OTU_4447 0.837 0.000461 *** |
| OTU_6510 0.837 0.000461 *** |
| OTU_6694 0.837 0.000461 *** |
| OTU_4495 0.837 0.000461 *** |
| OTU_4295 0.837 0.000847 *** |
| OTU_9262 0.837 0.000253 *** |
| OTU_5424 0.837 0.000847 *** |
| OTU_7053 0.837 0.000253 *** |
| OTU_8121 0.837 0.000461 *** |
| OTU_9014 0.837 0.001051 ** |
| OTU_5413 0.837 0.000847 *** |
| OTU_4821 0.837 0.000253 *** |
| OTU_4776 0.837 0.000461 *** |
| OTU_5513 0.837 0.000847 *** |
| OTU_8374 0.837 0.000461 *** |
| OTU_5562 0.837 0.000253 *** |
| OTU_8241 0.837 0.000658 *** |
| OTU_6859 0.837 0.000461 *** |
| OTU_8784 0.837 0.000658 *** |
| OTU_2111 0.833 0.000847 *** |
| OTU_1728 0.832 0.000658 *** |
| OTU_2231 0.832 0.001051 ** |
| OTU_1553 0.831 0.001440 ** |
| OTU_4640 0.831 0.001255 ** |
| OTU_9250 0.831 0.000847 *** |
| OTU_2946 0.831 0.001051 ** |
| OTU_6165 0.831 0.000658 *** |
| OTU_2412 0.831 0.000847 *** |
| OTU_3364 0.831 0.001594 ** |
| OTU_398 0.830 0.001779 ** |
| OTU_1740 0.830 0.000253 *** |
| OTU_2775 0.830 0.002061 ** |
| OTU_3205 0.830 0.000253 *** |
| OTU_3837 0.829 0.001594 ** |
| OTU_4391 0.829 0.001051 ** |
| OTU_11381 0.829 0.000253 *** |
| OTU_2459 0.828 0.000461 *** |
| OTU_2801 0.828 0.007865 ** |
| OTU_5525 0.828 0.000253 *** |
| OTU_1896 0.827 0.002729 ** |
| OTU_3618 0.827 0.000658 *** |
| OTU_3654 0.827 0.001594 ** |
| OTU_4572 0.827 0.004931 ** |
| OTU_2635 0.826 0.002549 ** |
| OTU_1434 0.826 0.000847 *** |
| OTU_4631 0.826 0.000658 *** |
| OTU_3206 0.825 0.000658 *** |
| OTU_3733 0.825 0.000658 *** |
| OTU_12131 0.825 0.000847 *** |
| OTU_3414 0.825 0.001779 ** |
| OTU_2767 0.825 0.000253 *** |
| OTU_715 0.824 0.000461 *** |
| OTU_1080 0.824 0.000461 *** |
| OTU_9133 0.824 0.001255 ** |
| OTU_5727 0.823 0.001440 ** |
| OTU_775 0.823 0.001051 ** |
| OTU_9337 0.823 0.001255 ** |
| OTU_10554 0.823 0.000847 *** |
| OTU_4617 0.823 0.001779 ** |
| OTU_2931 0.822 0.000253 *** |
| OTU_2912 0.822 0.001931 ** |
| OTU_3518 0.821 0.001440 ** |
| OTU_77 0.820 0.000658 *** |
| OTU_6096 0.820 0.000658 *** |
| OTU_2532 0.819 0.000253 *** |
| OTU_599 0.819 0.005272 ** |
| OTU_7985 0.819 0.001255 ** |
| OTU_1523 0.819 0.001594 ** |
| OTU_8444 0.815 0.001594 ** |
| OTU_5644 0.815 0.001255 ** |
| OTU_5011 0.815 0.000847 *** |
| OTU_2703 0.814 0.001440 ** |
| OTU_3433 0.813 0.001931 ** |
| OTU_3080 0.813 0.001440 ** |
| OTU_4419 0.812 0.001779 ** |
| OTU_878 0.812 0.003394 ** |
| OTU_2846 0.812 0.000658 *** |
| OTU_2799 0.812 0.003223 ** |
| OTU_4155 0.810 0.001779 ** |
| OTU_7371 0.810 0.002061 ** |
| OTU_4958 0.809 0.001931 ** |
| OTU_11989 0.808 0.001779 ** |
| OTU_6347 0.804 0.002061 ** |
| OTU_5619 0.803 0.001594 ** |
| OTU_3759 0.803 0.001440 ** |
| OTU_678 0.801 0.001051 ** |
| OTU_2733 0.800 0.002380 ** |
| OTU_10819 0.798 0.000847 *** |
| OTU_3630 0.791 0.001594 ** |
| OTU_9926 0.791 0.001779 ** |
| OTU_5712 0.789 0.004587 ** |
| OTU_4697 0.789 0.004931 ** |
| OTU_4684 0.785 0.006114 ** |
| OTU_9018 0.783 0.001931 ** |
| OTU_11316 0.783 0.002380 ** |
| OTU_2718 0.783 0.003716 ** |
| OTU_7454 0.783 0.001440 ** |
| OTU_3073 0.781 0.002549 ** |
| OTU_6007 0.779 0.002235 ** |
| OTU_3649 0.776 0.001931 ** |
| OTU_96 0.775 0.002061 ** |
| OTU_782 0.775 0.002380 ** |
| OTU_908 0.775 0.002380 ** |
| OTU_223 0.775 0.001931 ** |
| OTU_11080 0.775 0.001255 ** |
| OTU_4662 0.775 0.001931 ** |
| OTU_885 0.775 0.002235 ** |
| OTU_1169 0.775 0.003716 ** |
| OTU_541 0.775 0.003716 ** |
| OTU_1393 0.775 0.002380 ** |
| OTU_4685 0.775 0.002380 ** |
| OTU_3685 0.775 0.002061 ** |
| OTU_539 0.775 0.002380 ** |
| OTU_769 0.775 0.001931 ** |
| OTU_2699 0.775 0.002061 ** |
| OTU_5029 0.775 0.001931 ** |
| OTU_9270 0.775 0.002061 ** |
| OTU_958 0.775 0.002729 ** |
| OTU_9565 0.775 0.002380 ** |
| OTU_683 0.775 0.002549 ** |
| OTU_1429 0.775 0.002235 ** |
| OTU_3329 0.775 0.001594 ** |
| OTU_1906 0.775 0.001931 ** |
| OTU_2939 0.775 0.002380 ** |
| OTU_6372 0.775 0.002380 ** |
| OTU_2662 0.775 0.001440 ** |
| OTU_7190 0.775 0.001931 ** |
| OTU_6329 0.775 0.001594 ** |
| OTU_4346 0.775 0.001594 ** |
| OTU_4943 0.775 0.001594 ** |
| OTU_7897 0.775 0.002380 ** |
| OTU_5329 0.775 0.002894 ** |
| OTU_7036 0.775 0.003716 ** |
| OTU_10992 0.775 0.002380 ** |
| OTU_2560 0.775 0.003394 ** |
| OTU_2776 0.775 0.002061 ** |
| OTU_1426 0.775 0.003049 ** |
| OTU_1115 0.775 0.001931 ** |
| OTU_2881 0.775 0.002549 ** |
| OTU_914 0.775 0.002061 ** |
| OTU_4501 0.775 0.002380 ** |
| OTU_1614 0.775 0.002061 ** |
| OTU_1176 0.775 0.002235 ** |
| OTU_791 0.775 0.002380 ** |
| OTU_5241 0.775 0.001779 ** |
| OTU_2694 0.775 0.002061 ** |
| OTU_9453 0.775 0.001594 ** |
| OTU_2761 0.775 0.002235 ** |
| OTU_7610 0.775 0.002061 ** |
| OTU_1372 0.775 0.001931 ** |
| OTU_2750 0.775 0.002061 ** |
| OTU_10721 0.775 0.001594 ** |
| OTU_1886 0.775 0.001594 ** |
| OTU_4891 0.775 0.002061 ** |
| OTU_2486 0.775 0.001931 ** |
| OTU_4122 0.775 0.001594 ** |
| OTU_3061 0.775 0.002061 ** |
| OTU_2367 0.775 0.001594 ** |
| OTU_3161 0.775 0.002549 ** |
| OTU_6403 0.775 0.003716 ** |
| OTU_3134 0.775 0.001440 ** |
| OTU_11181 0.775 0.002549 ** |
| OTU_4196 0.775 0.002061 ** |
| OTU_1639 0.775 0.002380 ** |
| OTU_10377 0.775 0.002061 ** |
| OTU_4724 0.775 0.003049 ** |
| OTU_4568 0.775 0.002894 ** |
| OTU_4907 0.775 0.002380 ** |
| OTU_4163 0.775 0.003716 ** |
| OTU_10470 0.775 0.002061 ** |
| OTU_2792 0.775 0.001440 ** |
| OTU_12093 0.775 0.002061 ** |
| OTU_2715 0.775 0.001440 ** |
| OTU_5184 0.775 0.001931 ** |
| OTU_6887 0.775 0.003716 ** |
| OTU_5691 0.775 0.002061 ** |
| OTU_4254 0.775 0.002061 ** |
| OTU_11053 0.775 0.003394 ** |
| OTU_6188 0.775 0.001440 ** |
| OTU_3942 0.775 0.001594 ** |
| OTU_5813 0.775 0.002380 ** |
| OTU_12287 0.775 0.003049 ** |
| OTU_6579 0.775 0.002549 ** |
| OTU_4430 0.775 0.001440 ** |
| OTU_4770 0.775 0.002061 ** |
| OTU_3732 0.775 0.001594 ** |
| OTU_4625 0.775 0.001779 ** |
| OTU_10530 0.775 0.002380 ** |
| OTU_11526 0.775 0.002061 ** |
| OTU_7966 0.775 0.002061 ** |
| OTU_3575 0.775 0.001440 ** |
| OTU_7210 0.775 0.002894 ** |
| OTU_1491 0.775 0.002061 ** |
| OTU_4174 0.775 0.003049 ** |
| OTU_5098 0.775 0.001931 ** |
| OTU_4403 0.775 0.001440 ** |
| OTU_6827 0.775 0.017980 * |
| OTU_11419 0.775 0.002380 ** |
| OTU_6452 0.775 0.002061 ** |
| OTU_10496 0.775 0.001594 ** |
| OTU_4945 0.775 0.002894 ** |
| OTU_2136 0.775 0.002894 ** |
| OTU_3919 0.775 0.001931 ** |
| OTU_7416 0.775 0.001594 ** |
| OTU_3556 0.775 0.001440 ** |
| OTU_8514 0.775 0.002549 ** |
| OTU_8410 0.775 0.001931 ** |
| OTU_2578 0.775 0.001440 ** |
| OTU_3506 0.775 0.003716 ** |
| OTU_2545 0.775 0.001931 ** |
| OTU_5359 0.775 0.001594 ** |
| OTU_12146 0.775 0.003223 ** |
| OTU_4555 0.775 0.003716 ** |
| OTU_1789 0.775 0.002380 ** |
| OTU_4515 0.775 0.002061 ** |
| OTU_5990 0.775 0.001931 ** |
| OTU_1754 0.775 0.001440 ** |
| OTU_4335 0.775 0.003716 ** |
| OTU_5111 0.775 0.002380 ** |
| OTU_4044 0.775 0.002061 ** |
| OTU_5890 0.775 0.003049 ** |
| OTU_4503 0.775 0.001594 ** |
| OTU_5686 0.775 0.002061 ** |
| OTU_3469 0.775 0.001931 ** |
| OTU_5511 0.775 0.002380 ** |
| OTU_2386 0.775 0.002380 ** |
| OTU_5138 0.775 0.002061 ** |
| OTU_8019 0.775 0.002061 ** |
| OTU_3436 0.775 0.003049 ** |
| OTU_2821 0.775 0.001931 ** |
| OTU_3359 0.775 0.001594 ** |
| OTU_1592 0.775 0.002061 ** |
| OTU_3147 0.775 0.002549 ** |
| OTU_7986 0.775 0.002380 ** |
| OTU_4058 0.775 0.001931 ** |
| OTU_4935 0.775 0.001440 ** |
| OTU_3977 0.775 0.001931 ** |
| OTU_6633 0.775 0.001594 ** |
| OTU_5504 0.775 0.002380 ** |
| OTU_5918 0.775 0.002894 ** |
| OTU_7179 0.775 0.001931 ** |
| OTU_5405 0.775 0.002061 ** |
| OTU_5248 0.775 0.001594 ** |
| OTU_5242 0.775 0.001594 ** |
| OTU_7780 0.775 0.001594 ** |
| OTU_4123 0.775 0.003049 ** |
| OTU_4043 0.775 0.001594 ** |
| OTU_8022 0.775 0.001440 ** |
| OTU_4714 0.775 0.002061 ** |
| OTU_5263 0.775 0.002061 ** |
| OTU_5197 0.775 0.001440 ** |
| OTU_2909 0.775 0.001931 ** |
| OTU_4613 0.775 0.003049 ** |
| OTU_7574 0.775 0.001931 ** |
| OTU_4102 0.775 0.001255 ** |
| OTU_6715 0.775 0.002894 ** |
| OTU_6160 0.775 0.002235 ** |
| OTU_8842 0.775 0.001594 ** |
| OTU_9477 0.775 0.001594 ** |
| OTU_4564 0.775 0.001440 ** |
| OTU_8949 0.775 0.003049 ** |
| OTU_3226 0.775 0.002894 ** |
| OTU_3151 0.775 0.001594 ** |
| OTU_3505 0.775 0.001440 ** |
| OTU_5080 0.775 0.001440 ** |
| OTU_10813 0.775 0.001594 ** |
| OTU_11841 0.775 0.003223 ** |
| OTU_6008 0.775 0.002061 ** |
| OTU_6058 0.775 0.001779 ** |
| OTU_7943 0.775 0.001931 ** |
| OTU_4582 0.775 0.001594 ** |
| OTU_2918 0.775 0.002380 ** |
| OTU_5675 0.775 0.003716 ** |
| OTU_6483 0.775 0.001440 ** |
| OTU_5129 0.775 0.001594 ** |
| OTU_6790 0.775 0.003716 ** |
| OTU_3886 0.775 0.002380 ** |
| OTU_4106 0.775 0.002061 ** |
| OTU_4636 0.775 0.001440 ** |
| OTU_9547 0.775 0.003049 ** |
| OTU_3119 0.775 0.003716 ** |
| OTU_4561 0.775 0.001594 ** |
| OTU_3825 0.775 0.001594 ** |
| OTU_4511 0.775 0.001594 ** |
| OTU_5322 0.775 0.001594 ** |
| OTU_5210 0.775 0.002380 ** |
| OTU_8816 0.775 0.002380 ** |
| OTU_3544 0.775 0.002061 ** |
| OTU_6386 0.775 0.001594 ** |
| OTU_5381 0.775 0.001779 ** |
| OTU_7343 0.775 0.001931 ** |
| OTU_4614 0.775 0.001594 ** |
| OTU_7965 0.775 0.003049 ** |
| OTU_2268 0.775 0.003049 ** |
| OTU_4834 0.775 0.001440 ** |
| OTU_4439 0.775 0.001594 ** |
| OTU_3225 0.775 0.002061 ** |
| OTU_5494 0.775 0.003716 ** |
| OTU_8945 0.775 0.002061 ** |
| OTU_4904 0.775 0.001594 ** |
| OTU_8431 0.775 0.002380 ** |
| OTU_6729 0.775 0.002061 ** |
| OTU_7227 0.775 0.001594 ** |
| OTU_6535 0.775 0.002061 ** |
| OTU_4768 0.775 0.002235 ** |
| OTU_4773 0.775 0.001594 ** |
| OTU_4573 0.775 0.002549 ** |
| OTU_6249 0.775 0.002235 ** |
| OTU_8604 0.775 0.003049 ** |
| OTU_6698 0.775 0.001440 ** |
| OTU_5531 0.775 0.001931 ** |
| OTU_3915 0.775 0.001931 ** |
| OTU_7981 0.775 0.001931 ** |
| OTU_4421 0.775 0.002380 ** |
| OTU_5124 0.775 0.001594 ** |
| OTU_4298 0.775 0.002061 ** |
| OTU_5610 0.775 0.002061 ** |
| OTU_6186 0.775 0.002380 ** |
| OTU_9459 0.775 0.001931 ** |
| OTU_4317 0.775 0.002380 ** |
| OTU_1802 0.775 0.002549 ** |
| OTU_4817 0.775 0.003716 ** |
| OTU_6658 0.775 0.001594 ** |
| OTU_6570 0.775 0.001931 ** |
| OTU_3393 0.775 0.002380 ** |
| OTU_6619 0.775 0.001931 ** |
| OTU_6469 0.775 0.002061 ** |
| OTU_3851 0.775 0.001931 ** |
| OTU_7446 0.775 0.001931 ** |
| OTU_3221 0.775 0.001779 ** |
| OTU_9608 0.775 0.001594 ** |
| OTU_4965 0.775 0.001931 ** |
| OTU_9990 0.775 0.001440 ** |
| OTU_5393 0.775 0.002061 ** |
| OTU_3604 0.775 0.001594 ** |
| OTU_6829 0.775 0.002061 ** |
| OTU_3896 0.775 0.002061 ** |
| OTU_8346 0.775 0.002235 ** |
| OTU_3931 0.775 0.001594 ** |
| OTU_4828 0.775 0.002549 ** |
| OTU_10725 0.775 0.001594 ** |
| OTU_5126 0.775 0.003716 ** |
| OTU_5485 0.775 0.003049 ** |
| OTU_5973 0.775 0.002380 ** |
| OTU_5382 0.775 0.003049 ** |
| OTU_6544 0.775 0.002380 ** |
| OTU_7045 0.775 0.001594 ** |
| OTU_4873 0.775 0.001440 ** |
| OTU_2904 0.775 0.001440 ** |
| OTU_3308 0.775 0.002061 ** |
| OTU_3640 0.775 0.001440 ** |
| OTU_8246 0.775 0.002549 ** |
| OTU_5817 0.775 0.002235 ** |
| OTU_5160 0.775 0.001594 ** |
| OTU_5505 0.775 0.001594 ** |
| OTU_4686 0.775 0.001440 ** |
| OTU_6991 0.775 0.001594 ** |
| OTU_5717 0.775 0.001594 ** |
| OTU_2874 0.775 0.001594 ** |
| OTU_7029 0.775 0.002380 ** |
| OTU_4213 0.775 0.002380 ** |
| OTU_7535 0.775 0.002061 ** |
| OTU_4826 0.775 0.003049 ** |
| OTU_7508 0.775 0.002061 ** |
| OTU_6652 0.775 0.001931 ** |
| OTU_4355 0.775 0.002061 ** |
| OTU_10806 0.775 0.001931 ** |
| OTU_5805 0.775 0.001594 ** |
| OTU_6416 0.775 0.001440 ** |
| OTU_5865 0.775 0.001931 ** |
| OTU_5360 0.775 0.002380 ** |
| OTU_6270 0.775 0.003049 ** |
| OTU_5292 0.775 0.001440 ** |
| OTU_6043 0.775 0.002061 ** |
| OTU_4489 0.775 0.002061 ** |
| OTU_11544 0.775 0.002061 ** |
| OTU_9195 0.775 0.002061 ** |
| OTU_6152 0.775 0.001440 ** |
| OTU_7974 0.775 0.001931 ** |
| OTU_6503 0.775 0.002380 ** |
| OTU_4384 0.775 0.001931 ** |
| OTU_12061 0.775 0.003049 ** |
| OTU_8877 0.775 0.002061 ** |
| OTU_5075 0.775 0.001594 ** |
| OTU_7786 0.775 0.001779 ** |
| OTU_10398 0.775 0.002380 ** |
| OTU_8884 0.775 0.001051 ** |
| OTU_9973 0.775 0.001594 ** |
| OTU_11379 0.775 0.001931 ** |
| OTU_7039 0.775 0.001255 ** |
| OTU_4761 0.775 0.002061 ** |
| OTU_4919 0.775 0.002061 ** |
| OTU_6012 0.775 0.001594 ** |
| OTU_3820 0.775 0.001594 ** |
| OTU_5719 0.775 0.001779 ** |
| OTU_4846 0.775 0.002380 ** |
| OTU_4756 0.775 0.001931 ** |
| OTU_5978 0.775 0.002061 ** |
| OTU_4365 0.775 0.001594 ** |
| OTU_3180 0.775 0.002380 ** |
| OTU_4325 0.775 0.001255 ** |
| OTU_5240 0.775 0.001440 ** |
| OTU_11887 0.775 0.003716 ** |
| OTU_6380 0.775 0.003716 ** |
| OTU_7898 0.775 0.001594 ** |
| OTU_7022 0.775 0.001594 ** |
| OTU_6794 0.775 0.001931 ** |
| OTU_8658 0.775 0.001779 ** |
| OTU_3895 0.775 0.001931 ** |
| OTU_3656 0.775 0.002380 ** |
| OTU_8605 0.775 0.001594 ** |
| OTU_7735 0.775 0.002061 ** |
| OTU_6772 0.775 0.001594 ** |
| OTU_4977 0.775 0.002894 ** |
| OTU_8117 0.775 0.001931 ** |
| OTU_6740 0.775 0.002235 ** |
| OTU_5342 0.775 0.002061 ** |
| OTU_8619 0.775 0.003716 ** |
| OTU_9293 0.775 0.001594 ** |
| OTU_7142 0.775 0.003049 ** |
| OTU_12226 0.775 0.001931 ** |
| OTU_6498 0.775 0.001931 ** |
| OTU_6318 0.775 0.001594 ** |
| OTU_10719 0.775 0.003049 ** |
| OTU_5148 0.775 0.002061 ** |
| OTU_9496 0.775 0.002380 ** |
| OTU_6228 0.775 0.001594 ** |
| OTU_5291 0.775 0.002061 ** |
| OTU_3699 0.775 0.003049 ** |
| OTU_11496 0.775 0.002061 ** |
| OTU_7728 0.775 0.001779 ** |
| OTU_5395 0.775 0.001594 ** |
| OTU_8726 0.775 0.002061 ** |
| OTU_9513 0.775 0.002380 ** |
| OTU_6285 0.775 0.002061 ** |
| OTU_5276 0.775 0.002061 ** |
| OTU_5303 0.775 0.001594 ** |
| OTU_9203 0.775 0.001779 ** |
| OTU_5965 0.775 0.002380 ** |
| OTU_6566 0.775 0.002061 ** |
| OTU_8041 0.775 0.002894 ** |
| OTU_5508 0.775 0.003716 ** |
| OTU_4975 0.775 0.001931 ** |
| OTU_10760 0.775 0.001779 ** |
| OTU_4783 0.775 0.002061 ** |
| OTU_4895 0.775 0.002061 ** |
| OTU_6265 0.775 0.002894 ** |
| OTU_4726 0.775 0.002061 ** |
| OTU_7338 0.775 0.002061 ** |
| OTU_6965 0.775 0.002061 ** |
| OTU_5872 0.775 0.001594 ** |
| OTU_9142 0.775 0.002549 ** |
| OTU_7565 0.775 0.002061 ** |
| OTU_6871 0.775 0.003049 ** |
| OTU_6782 0.775 0.001931 ** |
| OTU_5971 0.775 0.001594 ** |
| OTU_5311 0.775 0.002894 ** |
| OTU_7545 0.775 0.003049 ** |
| OTU_7860 0.775 0.002061 ** |
| OTU_8008 0.775 0.002061 ** |
| OTU_5821 0.775 0.001931 ** |
| OTU_8529 0.775 0.002235 ** |
| OTU_6925 0.775 0.002894 ** |
| OTU_5703 0.775 0.003049 ** |
| OTU_11789 0.775 0.001594 ** |
| OTU_7350 0.775 0.001594 ** |
| OTU_10484 0.775 0.001931 ** |
| OTU_8611 0.775 0.001931 ** |
| OTU_1210 0.772 0.011562 * |
| OTU_1125 0.771 0.004413 ** |
| OTU_8344 0.771 0.007865 ** |
| OTU_3543 0.771 0.001779 ** |
| OTU_3182 0.771 0.002061 ** |
| OTU_11637 0.770 0.003223 ** |
| OTU_4455 0.770 0.007123 ** |
| OTU_2458 0.770 0.001440 ** |
| OTU_3278 0.770 0.001594 ** |
| OTU_2571 0.769 0.002380 ** |
| OTU_3424 0.769 0.003890 ** |
| OTU_974 0.769 0.002235 ** |
| OTU_2114 0.769 0.006114 ** |
| OTU_1824 0.769 0.001594 ** |
| OTU_5862 0.768 0.002235 ** |
| OTU_3207 0.767 0.005438 ** |
| OTU_5849 0.767 0.007123 ** |
| OTU_5370 0.766 0.003568 ** |
| OTU_5434 0.766 0.001779 ** |
| OTU_6866 0.766 0.004413 ** |
| OTU_6125 0.766 0.003716 ** |
| OTU_6728 0.766 0.003394 ** |
| OTU_2708 0.765 0.001594 ** |
| OTU_5048 0.765 0.005773 ** |
| OTU_11417 0.764 0.007600 ** |
| OTU_2795 0.764 0.005100 ** |
| OTU_2041 0.764 0.001931 ** |
| OTU_5534 0.764 0.001931 ** |
| OTU_3307 0.764 0.002061 ** |
| OTU_3739 0.764 0.007279 ** |
| OTU_2448 0.762 0.005773 ** |
| OTU_6259 0.762 0.005100 ** |
| OTU_5549 0.762 0.005100 ** |
| OTU_5787 0.762 0.003568 ** |
| OTU_7744 0.762 0.002061 ** |
| OTU_3440 0.761 0.004413 ** |
| OTU_2613 0.761 0.008849 ** |
| OTU_4687 0.761 0.004243 ** |
| OTU_5652 0.761 0.006794 ** |
| OTU_4608 0.761 0.001931 ** |
| OTU_5456 0.761 0.005773 ** |
| OTU_4070 0.760 0.005608 ** |
| OTU_5196 0.760 0.003716 ** |
| OTU_6204 0.760 0.005438 ** |
| OTU_7106 0.758 0.005438 ** |
| OTU_1830 0.758 0.006283 ** |
| OTU_4527 0.758 0.007979 ** |
| OTU_5611 0.758 0.005608 ** |
| OTU_2639 0.757 0.009583 ** |
| OTU_4185 0.756 0.004587 ** |
| OTU_6054 0.756 0.006283 ** |
| OTU_6352 0.756 0.006114 ** |
| OTU_5808 0.755 0.007600 ** |
| OTU_8105 0.755 0.002894 ** |
| OTU_4652 0.755 0.007279 ** |
| OTU_5785 0.755 0.005100 ** |
| OTU_5477 0.755 0.003223 ** |
| OTU_4838 0.754 0.004413 ** |
| OTU_246 0.753 0.008127 ** |
| OTU_5290 0.753 0.009099 ** |
| OTU_3934 0.753 0.002549 ** |
| OTU_5577 0.753 0.004931 ** |
| OTU_6573 0.753 0.008127 ** |
| OTU_5946 0.753 0.004760 ** |
| OTU_6868 0.751 0.007600 ** |
| OTU_4284 0.751 0.003568 ** |
| OTU_8062 0.751 0.005438 ** |
| OTU_3468 0.751 0.005773 ** |
| OTU_5050 0.751 0.010264 * |
| OTU_5195 0.751 0.005773 ** |
| OTU_6703 0.750 0.006961 ** |
| OTU_4802 0.750 0.008949 ** |
| OTU_6695 0.750 0.003394 ** |
| OTU_3263 0.748 0.007279 ** |
| OTU_2281 0.748 0.014115 * |
| OTU_9520 0.748 0.007600 ** |
| OTU_1307 0.748 0.038453 * |
| OTU_2093 0.748 0.007865 ** |
| OTU_2995 0.747 0.006961 ** |
| OTU_5392 0.746 0.009701 ** |
| OTU_7614 0.746 0.007600 ** |
| OTU_4437 0.745 0.004760 ** |
| OTU_135 0.744 0.007865 ** |
| OTU_9816 0.743 0.006114 ** |
| OTU_7413 0.743 0.009701 ** |
| OTU_4856 0.742 0.003568 ** |
| OTU_6303 0.742 0.012164 * |
| OTU_11349 0.742 0.009846 ** |
| OTU_1442 0.741 0.016337 * |
| OTU_5784 0.740 0.007445 ** |
| OTU_137 0.740 0.039766 * |
| OTU_3615 0.739 0.009089 ** |
| OTU_5798 0.739 0.006625 ** |
| OTU_6243 0.737 0.006455 ** |
| OTU_8856 0.737 0.007704 ** |
| OTU_2732 0.736 0.008409 ** |
| OTU_10917 0.736 0.008702 ** |
| OTU_5829 0.735 0.005608 ** |
| OTU_3661 0.733 0.008794 ** |
| OTU_5801 0.733 0.007123 ** |
| OTU_6443 0.725 0.012920 * |
| OTU_3912 0.725 0.011116 * |
| OTU_3133 0.721 0.007600 ** |
| OTU_3880 0.721 0.006794 ** |
| OTU_4263 0.720 0.013520 * |
| OTU_3546 0.720 0.008409 ** |
| OTU_6494 0.720 0.008566 ** |
| OTU_3900 0.719 0.014865 * |
| OTU_4051 0.717 0.032945 * |
| OTU_6828 0.717 0.008849 ** |
| OTU_3949 0.716 0.019618 * |
| OTU_120 0.713 0.016054 * |
| OTU_1471 0.707 0.009099 ** |
| OTU_1023 0.707 0.010114 * |
| OTU_1081 0.707 0.009099 ** |
| OTU_458 0.707 0.009099 ** |
| OTU_480 0.707 0.008849 ** |
| OTU_4148 0.707 0.009099 ** |
| OTU_142 0.707 0.008849 ** |
| OTU_922 0.707 0.009846 ** |
| OTU_169 0.707 0.008849 ** |
| OTU_1018 0.707 0.009304 ** |
| OTU_11854 0.707 0.008409 ** |
| OTU_8933 0.707 0.007704 ** |
| OTU_1636 0.707 0.007704 ** |
| OTU_2361 0.707 0.008949 ** |
| OTU_514 0.707 0.009501 ** |
| OTU_148 0.707 0.007979 ** |
| OTU_764 0.707 0.009304 ** |
| OTU_256 0.707 0.008849 ** |
| OTU_453 0.707 0.008409 ** |
| OTU_5586 0.707 0.007979 ** |
| OTU_6339 0.707 0.009099 ** |
| OTU_492 0.707 0.008794 ** |
| OTU_1706 0.707 0.008409 ** |
| OTU_4587 0.707 0.009583 ** |
| OTU_6076 0.707 0.009583 ** |
| OTU_11458 0.707 0.008409 ** |
| OTU_234 0.707 0.010114 * |
| OTU_3876 0.707 0.009099 ** |
| OTU_1110 0.707 0.008794 ** |
| OTU_6529 0.707 0.009099 ** |
| OTU_9440 0.707 0.008794 ** |
| OTU_693 0.707 0.009501 ** |
| OTU_1425 0.707 0.009099 ** |
| OTU_1805 0.707 0.008794 ** |
| OTU_5660 0.707 0.010386 * |
| OTU_9132 0.707 0.010114 * |
| OTU_5482 0.707 0.009099 ** |
| OTU_4939 0.707 0.008849 ** |
| OTU_4698 0.707 0.008849 ** |
| OTU_11096 0.707 0.008794 ** |
| OTU_3082 0.707 0.009304 ** |
| OTU_1343 0.707 0.007279 ** |
| OTU_4290 0.707 0.009099 ** |
| OTU_4217 0.707 0.009501 ** |
| OTU_3025 0.707 0.009089 ** |
| OTU_9038 0.707 0.009583 ** |
| OTU_3486 0.707 0.008409 ** |
| OTU_7140 0.707 0.007979 ** |
| OTU_4872 0.707 0.008794 ** |
| OTU_6718 0.707 0.009583 ** |
| OTU_3028 0.707 0.009423 ** |
| OTU_11535 0.707 0.010386 * |
| OTU_6527 0.707 0.007704 ** |
| OTU_5697 0.707 0.009304 ** |
| OTU_3650 0.707 0.006625 ** |
| OTU_813 0.707 0.006283 ** |
| OTU_8290 0.707 0.009099 ** |
| OTU_3419 0.707 0.008794 ** |
| OTU_5085 0.707 0.009099 ** |
| OTU_5777 0.707 0.008849 ** |
| OTU_11487 0.707 0.009099 ** |
| OTU_1468 0.707 0.009501 ** |
| OTU_5928 0.707 0.008849 ** |
| OTU_3416 0.707 0.008409 ** |
| OTU_7676 0.707 0.007979 ** |
| OTU_2401 0.707 0.008794 ** |
| OTU_1166 0.707 0.009982 ** |
| OTU_5323 0.707 0.009701 ** |
| OTU_2766 0.707 0.009701 ** |
| OTU_7376 0.707 0.009501 ** |
| OTU_2992 0.707 0.007704 ** |
| OTU_4736 0.707 0.007704 ** |
| OTU_3911 0.707 0.009583 ** |
| OTU_5179 0.707 0.009304 ** |
| OTU_11020 0.707 0.009846 ** |
| OTU_9451 0.707 0.008949 ** |
| OTU_4578 0.707 0.007704 ** |
| OTU_9788 0.707 0.008849 ** |
| OTU_4877 0.707 0.008849 ** |
| OTU_6862 0.707 0.009501 ** |
| OTU_4863 0.707 0.008409 ** |
| OTU_5490 0.707 0.009304 ** |
| OTU_1834 0.707 0.010386 * |
| OTU_6446 0.707 0.007445 ** |
| OTU_6197 0.707 0.008294 ** |
| OTU_4041 0.707 0.008702 ** |
| OTU_4518 0.707 0.009099 ** |
| OTU_2751 0.707 0.007704 ** |
| OTU_5774 0.707 0.008849 ** |
| OTU_11568 0.707 0.008949 ** |
| OTU_2600 0.707 0.008702 ** |
| OTU_5300 0.707 0.008849 ** |
| OTU_11768 0.707 0.010513 * |
| OTU_1919 0.707 0.009501 ** |
| OTU_4060 0.707 0.008949 ** |
| OTU_5602 0.707 0.007979 ** |
| OTU_7494 0.707 0.009501 ** |
| OTU_11794 0.707 0.009304 ** |
| OTU_5694 0.707 0.009701 ** |
| OTU_1765 0.707 0.009583 ** |
| OTU_2569 0.707 0.008794 ** |
| OTU_2969 0.707 0.007704 ** |
| OTU_6072 0.707 0.009701 ** |
| OTU_2202 0.707 0.007704 ** |
| OTU_5701 0.707 0.008702 ** |
| OTU_9482 0.707 0.009099 ** |
| OTU_8322 0.707 0.008702 ** |
| OTU_8147 0.707 0.009099 ** |
| OTU_8659 0.707 0.009099 ** |
| OTU_2176 0.707 0.007279 ** |
| OTU_2328 0.707 0.009501 ** |
| OTU_4505 0.707 0.009304 ** |
| OTU_6518 0.707 0.008794 ** |
| OTU_7880 0.707 0.008409 ** |
| OTU_3007 0.707 0.007979 ** |
| OTU_1460 0.707 0.009423 ** |
| OTU_12257 0.707 0.007979 ** |
| OTU_9505 0.707 0.008794 ** |
| OTU_6145 0.707 0.008127 ** |
| OTU_6520 0.707 0.008849 ** |
| OTU_8039 0.707 0.008949 ** |
| OTU_5025 0.707 0.009701 ** |
| OTU_5379 0.707 0.008794 ** |
| OTU_5902 0.707 0.007704 ** |
| OTU_4547 0.707 0.009235 ** |
| OTU_4851 0.707 0.009304 ** |
| OTU_8292 0.707 0.008949 ** |
| OTU_9515 0.707 0.008949 ** |
| OTU_3674 0.707 0.009304 ** |
| OTU_8812 0.707 0.009501 ** |
| OTU_8068 0.707 0.009423 ** |
| OTU_6113 0.707 0.008849 ** |
| OTU_5907 0.707 0.009501 ** |
| OTU_7159 0.707 0.008949 ** |
| OTU_1675 0.707 0.008794 ** |
| OTU_2836 0.707 0.008949 ** |
| OTU_3344 0.707 0.009304 ** |
| OTU_3807 0.707 0.009089 ** |
| OTU_6183 0.707 0.009423 ** |
| OTU_5873 0.707 0.009501 ** |
| OTU_7876 0.707 0.009099 ** |
| OTU_5781 0.707 0.007704 ** |
| OTU_4575 0.707 0.009099 ** |
| OTU_4156 0.707 0.008849 ** |
| OTU_4129 0.707 0.009099 ** |
| OTU_5631 0.707 0.008949 ** |
| OTU_6672 0.707 0.009099 ** |
| OTU_4446 0.707 0.009982 ** |
| OTU_3456 0.707 0.010513 * |
| OTU_3217 0.707 0.009583 ** |
| OTU_7681 0.707 0.008409 ** |
| OTU_4074 0.707 0.009304 ** |
| OTU_6766 0.707 0.007704 ** |
| OTU_4567 0.707 0.009089 ** |
| OTU_5779 0.707 0.009099 ** |
| OTU_5350 0.707 0.007979 ** |
| OTU_4510 0.707 0.007979 ** |
| OTU_8732 0.707 0.009583 ** |
| OTU_8177 0.707 0.007704 ** |
| OTU_7370 0.707 0.009583 ** |
| OTU_4231 0.707 0.009304 ** |
| OTU_8586 0.707 0.009583 ** |
| OTU_6737 0.707 0.008702 ** |
| OTU_4007 0.707 0.008702 ** |
| OTU_3542 0.707 0.009099 ** |
| OTU_11147 0.707 0.009583 ** |
| OTU_9201 0.707 0.009423 ** |
| OTU_9304 0.707 0.007704 ** |
| OTU_6624 0.707 0.008849 ** |
| OTU_6550 0.707 0.008849 ** |
| OTU_2347 0.707 0.009982 ** |
| OTU_5103 0.707 0.009501 ** |
| OTU_11288 0.707 0.008949 ** |
| OTU_10511 0.707 0.009099 ** |
| OTU_6581 0.707 0.009583 ** |
| OTU_6997 0.707 0.009235 ** |
| OTU_8643 0.707 0.008794 ** |
| OTU_4065 0.707 0.010513 * |
| OTU_5159 0.707 0.009099 ** |
| OTU_4487 0.707 0.009701 ** |
| OTU_5199 0.707 0.008849 ** |
| OTU_4779 0.707 0.009583 ** |
| OTU_7754 0.707 0.010386 * |
| OTU_10891 0.707 0.009583 ** |
| OTU_7598 0.707 0.010386 * |
| OTU_5385 0.707 0.007704 ** |
| OTU_7968 0.707 0.010386 * |
| OTU_11912 0.707 0.010513 * |
| OTU_7895 0.707 0.009099 ** |
| OTU_4146 0.707 0.009235 ** |
| OTU_3730 0.707 0.009099 ** |
| OTU_8208 0.707 0.009304 ** |
| OTU_7330 0.707 0.008949 ** |
| OTU_2851 0.707 0.008794 ** |
| OTU_11848 0.707 0.009501 ** |
| OTU_5953 0.707 0.009583 ** |
| OTU_3356 0.707 0.009304 ** |
| OTU_7131 0.707 0.009099 ** |
| OTU_3446 0.707 0.009304 ** |
| OTU_4071 0.707 0.008127 ** |
| OTU_5667 0.707 0.008566 ** |
| OTU_5325 0.707 0.008849 ** |
| OTU_9614 0.707 0.008949 ** |
| OTU_7506 0.707 0.007979 ** |
| OTU_8326 0.707 0.009099 ** |
| OTU_5472 0.707 0.009501 ** |
| OTU_6621 0.707 0.009501 ** |
| OTU_1839 0.707 0.009304 ** |
| OTU_11131 0.707 0.008849 ** |
| OTU_4441 0.707 0.007704 ** |
| OTU_6061 0.707 0.009099 ** |
| OTU_2989 0.707 0.009304 ** |
| OTU_8230 0.707 0.009099 ** |
| OTU_9805 0.707 0.008794 ** |
| OTU_6205 0.707 0.009501 ** |
| OTU_7407 0.707 0.009583 ** |
| OTU_5880 0.707 0.008409 ** |
| OTU_5214 0.707 0.007704 ** |
| OTU_5626 0.707 0.010386 * |
| OTU_5396 0.707 0.008949 ** |
| OTU_11856 0.707 0.010114 * |
| OTU_5984 0.707 0.009583 ** |
| OTU_4554 0.707 0.008702 ** |
| OTU_5110 0.707 0.009099 ** |
| OTU_5285 0.707 0.008794 ** |
| OTU_5333 0.707 0.009583 ** |
| OTU_9719 0.707 0.010967 * |
| OTU_5778 0.707 0.008566 ** |
| OTU_12013 0.707 0.007704 ** |
| OTU_9538 0.707 0.008849 ** |
| OTU_9651 0.707 0.009099 ** |
| OTU_7401 0.707 0.009099 ** |
| OTU_11539 0.707 0.009099 ** |
| OTU_10785 0.707 0.008702 ** |
| OTU_6332 0.707 0.009304 ** |
| OTU_5922 0.707 0.009099 ** |
| OTU_10074 0.707 0.007445 ** |
| OTU_8457 0.707 0.008949 ** |
| OTU_6031 0.707 0.009304 ** |
| OTU_8186 0.707 0.007979 ** |
| OTU_8205 0.707 0.008409 ** |
| OTU_4849 0.707 0.009235 ** |
| OTU_5876 0.707 0.009423 ** |
| OTU_4893 0.707 0.008794 ** |
| OTU_6387 0.707 0.007123 ** |
| OTU_3601 0.707 0.007979 ** |
| OTU_3262 0.707 0.008409 ** |
| OTU_4928 0.707 0.008949 ** |
| OTU_11248 0.707 0.008949 ** |
| OTU_4901 0.707 0.007704 ** |
| OTU_7044 0.707 0.008702 ** |
| OTU_12274 0.707 0.009089 ** |
| OTU_4730 0.707 0.009982 ** |
| OTU_7220 0.707 0.008849 ** |
| OTU_4358 0.707 0.008127 ** |
| OTU_5827 0.707 0.009099 ** |
| OTU_6433 0.707 0.008849 ** |
| OTU_7188 0.707 0.009423 ** |
| OTU_7984 0.707 0.009501 ** |
| OTU_10611 0.707 0.008849 ** |
| OTU_9773 0.707 0.008794 ** |
| OTU_11705 0.707 0.009701 ** |
| OTU_5225 0.707 0.010114 * |
| OTU_5033 0.707 0.008409 ** |
| OTU_8687 0.707 0.008849 ** |
| OTU_12099 0.707 0.007979 ** |
| OTU_7644 0.707 0.007979 ** |
| OTU_11524 0.707 0.009304 ** |
| OTU_4721 0.707 0.009099 ** |
| OTU_5162 0.707 0.007979 ** |
| OTU_7764 0.707 0.009089 ** |
| OTU_10172 0.707 0.009501 ** |
| OTU_6597 0.707 0.010114 * |
| OTU_6616 0.707 0.008849 ** |
| OTU_7411 0.707 0.009304 ** |
| OTU_6101 0.707 0.008127 ** |
| OTU_7962 0.707 0.008702 ** |
| OTU_5576 0.707 0.009099 ** |
| OTU_8340 0.707 0.009701 ** |
| OTU_6257 0.707 0.008794 ** |
| OTU_11829 0.707 0.010513 * |
| OTU_9612 0.707 0.008849 ** |
| OTU_5620 0.707 0.009099 ** |
| OTU_7830 0.707 0.009423 ** |
| OTU_6283 0.707 0.008409 ** |
| OTU_5052 0.707 0.008794 ** |
| OTU_5796 0.707 0.009099 ** |
| OTU_11113 0.707 0.009701 ** |
| OTU_4878 0.707 0.009423 ** |
| OTU_8040 0.707 0.008849 ** |
| OTU_7335 0.707 0.009583 ** |
| OTU_6759 0.707 0.008566 ** |
| OTU_6646 0.707 0.009304 ** |
| OTU_6741 0.707 0.008949 ** |
| OTU_5168 0.707 0.008849 ** |
| OTU_9828 0.707 0.009423 ** |
| OTU_8504 0.707 0.009099 ** |
| OTU_6612 0.707 0.009304 ** |
| OTU_9419 0.707 0.006961 ** |
| OTU_5838 0.707 0.008409 ** |
| OTU_5573 0.707 0.007704 ** |
| OTU_7475 0.707 0.008794 ** |
| OTU_11742 0.707 0.007704 ** |
| OTU_5442 0.707 0.010114 * |
| OTU_5598 0.707 0.009235 ** |
| OTU_5932 0.707 0.009099 ** |
| OTU_7679 0.707 0.007979 ** |
| OTU_3882 0.707 0.007704 ** |
| OTU_10609 0.707 0.009423 ** |
| OTU_7006 0.707 0.008794 ** |
| OTU_3706 0.707 0.008409 ** |
| OTU_5223 0.707 0.007704 ** |
| OTU_11918 0.707 0.009501 ** |
| OTU_5274 0.707 0.009099 ** |
| OTU_7900 0.707 0.007123 ** |
| OTU_7265 0.707 0.009304 ** |
| OTU_6212 0.707 0.009423 ** |
| OTU_7835 0.707 0.009304 ** |
| OTU_11817 0.707 0.009501 ** |
| OTU_7277 0.707 0.008849 ** |
| OTU_7119 0.707 0.009089 ** |
| OTU_9438 0.707 0.009235 ** |
| OTU_10701 0.707 0.008849 ** |
| OTU_3185 0.707 0.007279 ** |
| OTU_7243 0.707 0.009501 ** |
| OTU_6489 0.707 0.009423 ** |
| OTU_9461 0.707 0.009304 ** |
| OTU_6778 0.707 0.008849 ** |
| OTU_5065 0.707 0.008949 ** |
| OTU_5361 0.707 0.009099 ** |
| OTU_6281 0.707 0.008127 ** |
| OTU_5064 0.707 0.009099 ** |
| OTU_10054 0.707 0.009583 ** |
| OTU_8126 0.707 0.009304 ** |
| OTU_7013 0.707 0.009501 ** |
| OTU_8302 0.707 0.009583 ** |
| OTU_7462 0.707 0.009304 ** |
| OTU_6909 0.707 0.008849 ** |
| OTU_10646 0.707 0.009304 ** |
| OTU_5951 0.707 0.008794 ** |
| OTU_4436 0.707 0.010386 * |
| OTU_6679 0.707 0.008849 ** |
| OTU_9996 0.707 0.010513 * |
| OTU_6684 0.707 0.007979 ** |
| OTU_8400 0.707 0.009099 ** |
| OTU_7251 0.707 0.009235 ** |
| OTU_7693 0.707 0.009099 ** |
| OTU_6476 0.707 0.007279 ** |
| OTU_5646 0.707 0.008949 ** |
| OTU_10569 0.707 0.009099 ** |
| OTU_6623 0.707 0.007979 ** |
| OTU_7266 0.707 0.009099 ** |
| OTU_8381 0.707 0.009501 ** |
| OTU_8012 0.707 0.008794 ** |
| OTU_5831 0.707 0.008949 ** |
| OTU_6201 0.707 0.007704 ** |
| OTU_7883 0.707 0.009089 ** |
| OTU_5776 0.707 0.009501 ** |
| OTU_8428 0.707 0.010513 * |
| OTU_7082 0.707 0.009701 ** |
| OTU_9514 0.707 0.009982 ** |
| OTU_9997 0.707 0.008409 ** |
| OTU_6879 0.707 0.008849 ** |
| OTU_9392 0.707 0.009583 ** |
| OTU_11931 0.707 0.009501 ** |
| OTU_8866 0.707 0.009501 ** |
| OTU_7351 0.707 0.010386 * |
| OTU_8558 0.707 0.008849 ** |
| OTU_7090 0.707 0.008127 ** |
| OTU_7247 0.707 0.009304 ** |
| OTU_6107 0.707 0.019758 * |
| OTU_5570 0.707 0.007979 ** |
| OTU_3473 0.701 0.013820 * |
| OTU_6569 0.701 0.009982 ** |
| OTU_6139 0.701 0.009583 ** |
| OTU_10360 0.700 0.015164 * |
| OTU_3591 0.700 0.024767 * |
| OTU_4264 0.699 0.015311 * |
| OTU_4465 0.697 0.016932 * |
| OTU_4034 0.697 0.027703 * |
| OTU_2312 0.697 0.019473 * |
| OTU_5906 0.697 0.007979 ** |
| OTU_7191 0.696 0.021961 * |
| OTU_5869 0.695 0.016786 * |
| OTU_7319 0.695 0.016054 * |
| OTU_6559 0.694 0.010386 * |
| OTU_1102 0.693 0.019758 * |
| OTU_3831 0.692 0.007704 ** |
| OTU_5051 0.692 0.027837 * |
| OTU_4387 0.692 0.027703 * |
| OTU_5552 0.691 0.021083 * |
| OTU_8269 0.690 0.021228 * |
| OTU_5384 0.689 0.014262 * |
| OTU_4999 0.689 0.020939 * |
| OTU_4574 0.689 0.023731 * |
| OTU_4813 0.688 0.009099 ** |
| OTU_7398 0.688 0.025810 * |
| OTU_1324 0.687 0.020939 * |
| OTU_3247 0.687 0.023144 * |
| OTU_7773 0.686 0.017230 * |
| OTU_4042 0.685 0.016196 * |
| OTU_3651 0.685 0.033600 * |
| OTU_9422 0.685 0.016196 * |
| OTU_832 0.684 0.031699 * |
| OTU_4431 0.683 0.021961 * |
| OTU_4702 0.683 0.018724 * |
| OTU_5868 0.683 0.020505 * |
| OTU_9839 0.681 0.013967 * |
| OTU_6645 0.681 0.023587 * |
| OTU_4705 0.678 0.041232 * |
| OTU_1713 0.674 0.022549 * |
| OTU_3711 0.674 0.021823 * |
| OTU_6224 0.674 0.027837 * |
| OTU_8674 0.671 0.016337 * |
| OTU_7607 0.671 0.026979 * |
| OTU_7305 0.671 0.026403 * |
| OTU_6539 0.671 0.022405 * |
| OTU_7 0.667 0.034874 * |
| OTU_3243 0.667 0.028128 * |
| OTU_4444 0.665 0.033261 * |
| OTU_6055 0.664 0.028252 * |
| OTU_7654 0.664 0.024324 * |
| OTU_6170 0.663 0.026545 * |
| OTU_4323 0.663 0.031336 * |
| OTU_11237 0.661 0.034072 * |
| OTU_4451 0.661 0.026837 * |
| OTU_1193 0.661 0.028252 * |
| OTU_5724 0.661 0.011414 * |
| OTU_6507 0.658 0.032835 * |
| OTU_7591 0.658 0.028252 * |
| OTU_6359 0.655 0.028543 * |
| OTU_7289 0.655 0.032098 * |
| OTU_6194 0.650 0.033600 * |
| OTU_3096 0.649 0.045494 * |
| OTU_5972 0.645 0.032945 * |
| OTU_42 0.632 0.031699 * |
| OTU_7989 0.632 0.032945 * |
| OTU_354 0.632 0.036715 * |
| OTU_2564 0.632 0.033048 * |
| OTU_131 0.632 0.033261 * |
| OTU_2650 0.632 0.033048 * |
| OTU_9896 0.632 0.034288 * |
| OTU_572 0.632 0.034644 * |
| OTU_6095 0.632 0.033318 * |
| OTU_7250 0.632 0.032835 * |
| OTU_8716 0.632 0.033048 * |
| OTU_7154 0.632 0.032338 * |
| OTU_7385 0.632 0.033048 * |
| OTU_1821 0.632 0.034072 * |
| OTU_2474 0.632 0.034188 * |
| OTU_1753 0.632 0.034493 * |
| OTU_2876 0.632 0.034493 * |
| OTU_6599 0.632 0.035157 * |
| OTU_5487 0.632 0.032945 * |
| OTU_3860 0.632 0.034478 * |
| OTU_3210 0.632 0.033665 * |
| OTU_8001 0.632 0.032945 * |
| OTU_401 0.632 0.033048 * |
| OTU_5832 0.632 0.033050 * |
| OTU_7501 0.632 0.033048 * |
| OTU_9640 0.632 0.032098 * |
| OTU_11550 0.632 0.034126 * |
| OTU_9089 0.632 0.033665 * |
| OTU_2575 0.632 0.033048 * |
| OTU_1246 0.632 0.033525 * |
| OTU_1314 0.632 0.033665 * |
| OTU_5659 0.632 0.034126 * |
| OTU_1044 0.632 0.032945 * |
| OTU_7611 0.632 0.033048 * |
| OTU_873 0.632 0.033665 * |
| OTU_1762 0.632 0.033952 * |
| OTU_924 0.632 0.033795 * |
| OTU_9192 0.632 0.033261 * |
| OTU_7476 0.632 0.034493 * |
| OTU_7005 0.632 0.032219 * |
| OTU_3457 0.632 0.032945 * |
| OTU_1645 0.632 0.033795 * |
| OTU_4545 0.632 0.032945 * |
| OTU_6248 0.632 0.034874 * |
| OTU_7957 0.632 0.033952 * |
| OTU_7033 0.632 0.033795 * |
| OTU_4183 0.632 0.033261 * |
| OTU_1151 0.632 0.033048 * |
| OTU_8232 0.632 0.033048 * |
| OTU_2686 0.632 0.032945 * |
| OTU_12053 0.632 0.033916 * |
| OTU_10707 0.632 0.033952 * |
| OTU_7143 0.632 0.033261 * |
| OTU_1196 0.632 0.033731 * |
| OTU_5764 0.632 0.034397 * |
| OTU_4700 0.632 0.033048 * |
| OTU_7477 0.632 0.034874 * |
| OTU_11212 0.632 0.033050 * |
| OTU_4463 0.632 0.032945 * |
| OTU_2010 0.632 0.033665 * |
| OTU_9624 0.632 0.034874 * |
| OTU_6626 0.632 0.033952 * |
| OTU_6892 0.632 0.032945 * |
| OTU_12270 0.632 0.034188 * |
| OTU_6470 0.632 0.034188 * |
| OTU_8386 0.632 0.034188 * |
| OTU_6328 0.632 0.033048 * |
| OTU_5425 0.632 0.032945 * |
| OTU_5308 0.632 0.036715 * |
| OTU_5458 0.632 0.034478 * |
| OTU_7761 0.632 0.033952 * |
| OTU_6010 0.632 0.033050 * |
| OTU_5891 0.632 0.032835 * |
| OTU_1796 0.632 0.033231 * |
| OTU_9155 0.632 0.033261 * |
| OTU_4372 0.632 0.033048 * |
| OTU_5202 0.632 0.034478 * |
| OTU_2399 0.632 0.034493 * |
| OTU_4141 0.632 0.032945 * |
| OTU_7723 0.632 0.033318 * |
| OTU_996 0.632 0.033048 * |
| OTU_2389 0.632 0.032945 * |
| OTU_6681 0.632 0.032835 * |
| OTU_5449 0.632 0.034874 * |
| OTU_10378 0.632 0.033146 * |
| OTU_6017 0.632 0.033231 * |
| OTU_3642 0.632 0.033731 * |
| OTU_7073 0.632 0.035157 * |
| OTU_2518 0.632 0.033795 * |
| OTU_10318 0.632 0.033048 * |
| OTU_5493 0.632 0.034493 * |
| OTU_4466 0.632 0.034288 * |
| OTU_7657 0.632 0.035157 * |
| OTU_10316 0.632 0.032945 * |
| OTU_5373 0.632 0.034126 * |
| OTU_11648 0.632 0.033731 * |
| OTU_3847 0.632 0.031699 * |
| OTU_3985 0.632 0.034288 * |
| OTU_6458 0.632 0.034188 * |
| OTU_2228 0.632 0.033525 * |
| OTU_4239 0.632 0.033048 * |
| OTU_6213 0.632 0.033146 * |
| OTU_4972 0.632 0.033050 * |
| OTU_3848 0.632 0.034126 * |
| OTU_3013 0.632 0.033048 * |
| OTU_9689 0.632 0.033048 * |
| OTU_7292 0.632 0.033048 * |
| OTU_3439 0.632 0.033048 * |
| OTU_12085 0.632 0.034188 * |
| OTU_9462 0.632 0.034644 * |
| OTU_3422 0.632 0.033525 * |
| OTU_9567 0.632 0.033600 * |
| OTU_1862 0.632 0.033048 * |
| OTU_6417 0.632 0.035107 * |
| OTU_6300 0.632 0.036715 * |
| OTU_6023 0.632 0.034188 * |
| OTU_6649 0.632 0.033048 * |
| OTU_3995 0.632 0.031699 * |
| OTU_5367 0.632 0.032945 * |
| OTU_4920 0.632 0.033048 * |
| OTU_5645 0.632 0.033952 * |
| OTU_11037 0.632 0.033050 * |
| OTU_7228 0.632 0.033600 * |
| OTU_3747 0.632 0.033048 * |
| OTU_9575 0.632 0.033048 * |
| OTU_3370 0.632 0.034874 * |
| OTU_3652 0.632 0.033318 * |
| OTU_11215 0.632 0.033261 * |
| OTU_6448 0.632 0.032945 * |
| OTU_9767 0.632 0.032945 * |
| OTU_5480 0.632 0.035157 * |
| OTU_5731 0.632 0.032945 * |
| OTU_4913 0.632 0.033600 * |
| OTU_2492 0.632 0.032945 * |
| OTU_7377 0.632 0.032835 * |
| OTU_4369 0.632 0.034478 * |
| OTU_2118 0.632 0.033048 * |
| OTU_6178 0.632 0.032945 * |
| OTU_9783 0.632 0.034874 * |
| OTU_3128 0.632 0.033048 * |
| OTU_6393 0.632 0.034188 * |
| OTU_6210 0.632 0.033048 * |
| OTU_6967 0.632 0.033952 * |
| OTU_10061 0.632 0.034644 * |
| OTU_9615 0.632 0.032835 * |
| OTU_7304 0.632 0.033048 * |
| OTU_7316 0.632 0.034288 * |
| OTU_4646 0.632 0.033318 * |
| OTU_4995 0.632 0.033525 * |
| OTU_5058 0.632 0.032945 * |
| OTU_4104 0.632 0.031699 * |
| OTU_6021 0.632 0.033048 * |
| OTU_4609 0.632 0.033525 * |
| OTU_6666 0.632 0.032945 * |
| OTU_3141 0.632 0.035157 * |
| OTU_3852 0.632 0.033665 * |
| OTU_2889 0.632 0.034478 * |
| OTU_2739 0.632 0.034478 * |
| OTU_11051 0.632 0.033048 * |
| OTU_5900 0.632 0.033048 * |
| OTU_7480 0.632 0.034188 * |
| OTU_4423 0.632 0.032945 * |
| OTU_7205 0.632 0.034493 * |
| OTU_4882 0.632 0.033048 * |
| OTU_6924 0.632 0.033525 * |
| OTU_8035 0.632 0.034493 * |
| OTU_5289 0.632 0.033048 * |
| OTU_9054 0.632 0.034188 * |
| OTU_4805 0.632 0.033048 * |
| OTU_4449 0.632 0.032945 * |
| OTU_3669 0.632 0.033048 * |
| OTU_9585 0.632 0.034644 * |
| OTU_6540 0.632 0.034874 * |
| OTU_4961 0.632 0.033048 * |
| OTU_8462 0.632 0.033048 * |
| OTU_10556 0.632 0.033795 * |
| OTU_7180 0.632 0.035282 * |
| OTU_7126 0.632 0.033231 * |
| OTU_8417 0.632 0.033048 * |
| OTU_10865 0.632 0.033048 * |
| OTU_6978 0.632 0.033795 * |
| OTU_6512 0.632 0.032945 * |
| OTU_7704 0.632 0.034493 * |
| OTU_8925 0.632 0.034188 * |
| OTU_4024 0.632 0.032945 * |
| OTU_5670 0.632 0.034874 * |
| OTU_2990 0.632 0.033050 * |
| OTU_5389 0.632 0.033048 * |
| OTU_5689 0.632 0.033050 * |
| OTU_12204 0.632 0.033600 * |
| OTU_4538 0.632 0.034188 * |
| OTU_9303 0.632 0.032338 * |
| OTU_7975 0.632 0.034126 * |
| OTU_7529 0.632 0.033048 * |
| OTU_7596 0.632 0.035107 * |
| OTU_11515 0.632 0.032945 * |
| OTU_5092 0.632 0.032945 * |
| OTU_8164 0.632 0.034874 * |
| OTU_7259 0.632 0.033146 * |
| OTU_4917 0.632 0.033048 * |
| OTU_7794 0.632 0.033048 * |
| OTU_6525 0.632 0.033050 * |
| OTU_8311 0.632 0.033146 * |
| OTU_4338 0.632 0.034126 * |
| OTU_3607 0.632 0.033048 * |
| OTU_7567 0.632 0.034126 * |
| OTU_7946 0.632 0.032945 * |
| OTU_6390 0.632 0.033048 * |
| OTU_6358 0.632 0.034874 * |
| OTU_9758 0.632 0.033261 * |
| OTU_6942 0.632 0.033048 * |
| OTU_7713 0.632 0.034644 * |
| OTU_4019 0.632 0.034188 * |
| OTU_2847 0.632 0.033665 * |
| OTU_5336 0.632 0.033261 * |
| OTU_7283 0.632 0.033795 * |
| OTU_7524 0.632 0.033048 * |
| OTU_7138 0.632 0.032945 * |
| OTU_9010 0.632 0.033048 * |
| OTU_3980 0.632 0.033261 * |
| OTU_8091 0.632 0.033048 * |
| OTU_9601 0.632 0.035157 * |
| OTU_6208 0.632 0.034493 * |
| OTU_6309 0.632 0.033048 * |
| OTU_6315 0.632 0.032945 * |
| OTU_7685 0.632 0.032098 * |
| OTU_11146 0.632 0.032945 * |
| OTU_6628 0.632 0.033048 * |
| OTU_6792 0.632 0.033048 * |
| OTU_4309 0.632 0.034126 * |
| OTU_4796 0.632 0.033048 * |
| OTU_6745 0.632 0.034188 * |
| OTU_3795 0.632 0.034644 * |
| OTU_6472 0.632 0.031056 * |
| OTU_6162 0.632 0.033525 * |
| OTU_9202 0.632 0.032945 * |
| OTU_7342 0.632 0.032945 * |
| OTU_6966 0.632 0.034644 * |
| OTU_5999 0.632 0.034188 * |
| OTU_10697 0.632 0.032338 * |
| OTU_7487 0.632 0.034188 * |
| OTU_8755 0.632 0.032945 * |
| OTU_5491 0.632 0.033048 * |
| OTU_7743 0.632 0.033795 * |
| OTU_6081 0.632 0.034493 * |
| OTU_5063 0.632 0.033048 * |
| OTU_5141 0.632 0.033525 * |
| OTU_6855 0.632 0.033731 * |
| OTU_8355 0.632 0.033048 * |
| OTU_6714 0.632 0.034188 * |
| OTU_7412 0.632 0.034188 * |
| OTU_11571 0.632 0.032945 * |
| OTU_5149 0.632 0.033916 * |
| OTU_6808 0.632 0.033048 * |
| OTU_9078 0.632 0.034874 * |
| OTU_9918 0.632 0.034644 * |
| OTU_9633 0.632 0.033048 * |
| OTU_9607 0.632 0.033318 * |
| OTU_5519 0.632 0.033048 * |
| OTU_9047 0.632 0.034644 * |
| OTU_4737 0.632 0.033952 * |
| OTU_7423 0.632 0.033146 * |
| OTU_7206 0.632 0.033318 * |
| OTU_11014 0.632 0.033048 * |
| OTU_8568 0.632 0.032835 * |
| OTU_6274 0.632 0.034644 * |
| OTU_10640 0.632 0.032219 * |
| OTU_11099 0.632 0.033318 * |
| OTU_9092 0.632 0.032945 * |
| OTU_4968 0.632 0.033048 * |
| OTU_4758 0.632 0.033731 * |
| OTU_7192 0.632 0.034874 * |
| OTU_5848 0.632 0.033318 * |
| OTU_5633 0.632 0.033050 * |
| OTU_8990 0.632 0.033231 * |
| OTU_7732 0.632 0.034397 * |
| OTU_3763 0.632 0.033318 * |
| OTU_10066 0.632 0.033731 * |
| OTU_9135 0.632 0.033231 * |
| OTU_7914 0.632 0.033048 * |
| OTU_6262 0.632 0.033731 * |
| OTU_5221 0.632 0.033048 * |
| OTU_6366 0.632 0.033050 * |
| OTU_8330 0.632 0.033050 * |
| OTU_5962 0.632 0.034644 * |
| OTU_8955 0.632 0.034493 * |
| OTU_7133 0.632 0.033048 * |
| OTU_6878 0.632 0.035107 * |
| OTU_7270 0.632 0.034188 * |
| OTU_7563 0.632 0.032098 * |
| OTU_8818 0.632 0.032945 * |
| OTU_10312 0.632 0.033050 * |
| OTU_6800 0.632 0.032098 * |
| OTU_6003 0.632 0.034493 * |
| OTU_11253 0.632 0.033048 * |
| OTU_6394 0.632 0.033048 * |
| OTU_6771 0.632 0.033050 * |
| OTU_8335 0.632 0.033048 * |
| OTU_6413 0.632 0.033600 * |
| OTU_6699 0.632 0.035107 * |
| OTU_10896 0.632 0.035157 * |
| OTU_11865 0.632 0.033048 * |
| OTU_7544 0.632 0.033048 * |
| OTU_8939 0.632 0.032764 * |
| OTU_10873 0.632 0.034874 * |
| OTU_6669 0.632 0.033261 * |
| OTU_7336 0.632 0.033048 * |
| OTU_8072 0.632 0.032945 * |
| OTU_8847 0.632 0.034188 * |
| OTU_6471 0.632 0.032945 * |
| OTU_7157 0.632 0.032945 * |
| OTU_10285 0.632 0.034493 * |
| OTU_6707 0.632 0.033050 * |
| OTU_5814 0.632 0.033048 * |
| OTU_8380 0.632 0.033261 * |
| OTU_6592 0.632 0.033048 * |
| OTU_6554 0.632 0.032945 * |
| OTU_7930 0.632 0.034397 * |
| OTU_7602 0.632 0.033231 * |
| OTU_6317 0.632 0.033048 * |
| OTU_8685 0.632 0.033048 * |
| OTU_7091 0.632 0.032764 * |
| OTU_7124 0.632 0.032835 * |
| OTU_8507 0.632 0.033048 * |
| OTU_11140 0.632 0.034874 * |
| OTU_6501 0.632 0.034188 * |
| OTU_10308 0.632 0.033048 * |
| OTU_5324 0.632 0.031056 * |
| OTU_11563 0.632 0.034644 * |
| OTU_7838 0.632 0.032945 * |
| OTU_8079 0.632 0.035157 * |
| OTU_6466 0.632 0.033665 * |
| OTU_10899 0.632 0.035157 * |
| OTU_11762 0.632 0.034188 * |
| OTU_8352 0.632 0.034874 * |
| OTU_10173 0.632 0.032945 * |
| OTU_6586 0.632 0.034188 * |
| OTU_8813 0.632 0.033952 * |
| OTU_6683 0.632 0.033048 * |
| OTU_11935 0.632 0.033731 * |
| OTU_7886 0.632 0.032945 * |
| OTU_6985 0.632 0.034874 * |
| OTU_6724 0.632 0.032945 * |
| OTU_8000 0.632 0.032945 * |
| OTU_10257 0.632 0.034874 * |
| OTU_5188 0.632 0.031699 * |
| OTU_8517 0.632 0.033952 * |
| OTU_7924 0.632 0.033952 * |
| OTU_24 0.627 0.044835 * |
| OTU_5421 0.619 0.033048 * |
| OTU_2734 0.619 0.049628 * |
| OTU_2322 0.617 0.032945 * |
| OTU_6331 0.614 0.031699 * |
| OTU_5097 0.612 0.033050 * |
| OTU_6989 0.612 0.038711 * |
| OTU_8371 0.612 0.031615 * |
| OTU_7041 0.611 0.033048 * |
| OTU_5914 0.608 0.032945 * |
| OTU_5855 0.607 0.034644 * |
| OTU_11202 0.592 0.034874 * |
|  |
| Group Stipe #sps. 2 |
| stat p.value |
| OTU_4709 0.749 0.0119 * |
| OTU_402 0.683 0.0184 * |
|  |
| Group Pileus+Soil #sps. 5 |
| stat p.value |
| OTU_362 0.948 0.0315 * |
| OTU_2182 0.887 0.0392 * |
| OTU_353 0.862 0.0341 * |
| OTU_1272 0.742 0.0359 * |
| OTU_1651 0.707 0.0355 * |
|  |
| Group Pileus+Stipe #sps. 4 |
| stat p.value |
| OTU_50 0.999 0.000253 *** |
| OTU_2352 0.998 0.003394 ** |
| OTU_79 0.982 0.000253 *** |
| OTU_5515 0.904 0.011414 * |
|  |
| Group Soil+Stipe #sps. 16 |
| stat p.value |
| OTU_956 0.997 0.000253 *** |
| OTU_320 0.965 0.002235 ** |
| OTU_147 0.931 0.003890 ** |
| OTU_63 0.917 0.000658 *** |
| OTU_800 0.886 0.003716 ** |
| OTU_802 0.879 0.002894 ** |
| OTU_645 0.866 0.005438 ** |
| OTU_1876 0.813 0.016054 * |
| OTU_5386 0.806 0.009583 ** |
| OTU_4117 0.775 0.013520 * |
| OTU_5766 0.774 0.049507 * |
| OTU_254 0.768 0.042967 * |
| OTU_905 0.761 0.031336 * |
| OTU_5575 0.756 0.033048 * |
| OTU_4532 0.742 0.027703 * |
| OTU_1725 0.742 0.032219 * |
| --- |
| Signif. codes: 0 ‚Äò***‚Äô 0.001 ‚Äò**‚Äô 0.01 ‚Äò*‚Äô 0.05 ‚Äò.‚Äô 0.1 ‚Äò ‚Äô 1 |
